# Disease-Tailored Brief Intervention for Alcohol Use Among Youths With Chronic Medical Conditions: A Secondary Analysis of a Randomized Clinical Trial

**DOI:** 10.1001/jamanetworkopen.2024.19858

**Published:** 2024-07-10

**Authors:** Elissa R. Weitzman, Machiko Minegishi, Fatma Dedeoglu, Laurie N. Fishman, Katharine C. Garvey, Lauren E. Wisk, Sharon Levy

**Affiliations:** 1 Division of Addiction Medicine, Boston Children’s Hospital, Boston, Massachusetts; 2 Department of Pediatrics, Harvard Medical School, Boston, Massachusetts; 3 Rheumatology Program, Division of Immunology, Boston Children’s Hospital, Boston, Massachusetts; 4 Division of Gastroenterology, Boston Children’s Hospital, Boston, Massachusetts; 5 Division of Endocrinology, Boston Children’s Hospital, Boston, Massachusetts; 6 Division of General Internal Medicine and Health Services Research, David Geffen School of Medicine at the University of California, Los Angeles

## Abstract

**Importance:**

In the US, 25% of youths have a chronic medical condition (CMC). Alcohol use is prevalent among youths with a CMC and is associated with treatment nonadherence, simultaneous exposure to contraindicated medications, poor self-care, and elevated rates of progression to heavy and problem use by young adulthood. Preventive interventions targeting these youths are scarce and lack evidence about longer-term risk-stratified effects.

**Objective:**

To evaluate the 12-month effects of a preventive intervention for alcohol use among youths with a CMC reporting baseline no or low-risk alcohol use vs high-risk alcohol use, testing the hypothesis of no difference.

**Design, Setting, and Participants:**

This presepecified secondary analysis used data from a 2-group, parallel randomized clinical trial of the Take Good Care (TGC) intervention. Convenience samples of youths (aged 14-18 years) with a CMC, such as type 1 diabetes, juvenile idiopathic arthritis, or inflammatory bowel disease, were randomly assigned to the intervention or treatment as usual (TAU) between May 11, 2017, and November 20, 2018, and were followed up for up to 12 months. High-risk alcohol use was defined as heavy episodic (binge) alcohol use in the past 3 months and alcohol-related blackouts, injuries, vomiting, or emergency department visits in the past 12 months. Data were analyzed from September 21, 2023, to February 3, 2024.

**Interventions:**

The self-administered, tablet computer–based TGC intervention was developed with patient and expert input, and it delivers disease-tailored psychoeducational content about the effects of alcohol use on overall health, disease processes, and treatment safety and efficacy.

**Main Outcomes and Measures:**

The main outcome was self-reported frequency of alcohol use (in days) over the past 3 months, measured by a single validated question. Maximum likelihood methods incorporating all available data were used assuming data missing at random.

**Results:**

The trial included 451 participants (229 female youths [50.8%]), with a mean (SD) age of 16.0 (1.4) years. Of these youths, 410 (90.9%) participated in the 12-month follow-up. At baseline, 52 youths (11.5%) reported high-risk alcohol use. Among participants with high-risk alcohol use, the observed mean (SD) frequency of alcohol use from baseline to the 12-month follow-up decreased in the intervention group (from 6.3 [4.6] to 4.9 [4.3] days) and increased in the TAU group (from 5.5 [4.9] to 9.0 [5.8] days), with an adjusted relative rate ratio of 0.60 (95% CI, 0.38 to 0.94). There were no group differences among youths reporting no or low-risk alcohol use.

**Conclusions and Relevance:**

In this trial of a brief chronic illness–tailored preventive intervention, medically vulnerable youths with a high risk of alcohol use and harm decreased alcohol use. These findings support the use of a personalized preventive intervention with this group.

**Trial Registration:**

ClinicalTrials.gov Identifier: NCT02803567

## Key Points

**Question:**

Does exposure to a brief, self-administered, disease-tailored preventive intervention lead to reduced alcohol use after 12 months among youths with a chronic medical condition (CMC)?

**Findings:**

This secondary analysis of a randomized clinical trial used data from 451 youths with a CMC. Among youths reporting high-risk alcohol use at baseline, there was a 40% relative reduction in alcohol use frequency among youths who received the intervention vs treatment as usual; no difference was observed among youths reporting no or low-risk use at baseline.

**Meaning:**

The intervention reduced alcohol use among medically vulnerable youths reporting high-risk use, underscoring the value of using personalized prevention strategies and investigating effect heterogeneities over long periods.

## Teaser

This secondary analysis of a randomized clinical trial examined the effects of a preventive intervention for alcohol use over 12 months among youths with a chronic medical condition.

## Introduction

In the US, 1 in 4 youths (25%) have a chronic medical condition (CMC)^1^ and are acutely vulnerable to the negative effects of alcohol and other substance use. Poor physical health, psychosocial distress (eg, depression and anxiety), pain, and other factors are associated with alcohol use by youths with a CMC.^2,3,4^ In addition, some youths with a CMC may use alcohol or other substances to address symptoms, treatment side effects, and distress.^3,4^ As a group, youths with a CMC have similar rates of alcohol and other substance use as other youths in early adolescence, but they are more likely to progress to heavy and problem use by young adulthood.^5^ Like their peers, youths with a CMC who use alcohol have an increased risk of accidents, injury, school failure, and other problems, including poor mental health related to alcohol use^6^; they also face unique and potentially grave risks for medical complications and disease exacerbations. Compared with youths with a CMC who do not use alcohol, those who do report nearly twice the odds of regular treatment nonadherence^7^; the majority (86.4%) take medications with alcohol use contraindications, and more than one-third of these youths (35.4%) use alcohol.^8^

For youths with a CMC, living with a chronic illness requires juggling self-care, disease management, frequent health care visits, and decision-making regarding health behaviors. Examples of these behaviors include whether to use alcohol and other substances while endeavoring to engage in routine social and academic activities. Yet there are few preventive interventions targeting this group’s alcohol use, despite their unique health concerns.^2,5,9,10,11,12,13^ In a prior study of an experimental evaluation of a brief, theory-based, self-administered chronic illness–tailored preventive intervention called Take Good Care (TGC), researchers observed favorable effects of the intervention among youths with a CMC who viewed it compared with those who received treatment as usual (TAU) on knowledge about alcohol’s adverse effects on health and disease status, intolerance of risks related to alcohol use and, among female youths, reductions in the frequency of use.^11^ Additionally, a comparative effectiveness trial of the TGC intervention delivered to adolescents and young adults with type 1 diabetes in colleges throughout the US reported favorable effects on rates of heavy episodic or binge drinking at the 2-week follow-up.^14^

Clinical trials of preventive interventions targeting alcohol use can reveal effect heterogeneities by personal sociodemographic characteristics or alcohol use history.^11,12,13,15,16^ Youths who have experience with heavy or harmful alcohol use may discount messages from a self-administered brief intervention.^17^ Alternatively, youths who have developed a severe alcohol use disorder may need ongoing support to attain and sustain behavior change. However, youths who have prior negative experiences with heavy alcohol use and associated harms but have not developed habitual use may be motivated and able to reduce their use over time, given exposure to personally salient information that engages them around negative experiences they may wish to avoid repeating. Relatively long follow-up periods may be necessary to document change in alcohol use behaviors among adolescents following exposure to a preventive intervention, given the sporadic nature of adolescent alcohol use.^18^ Longer-term follow-up periods may also provide insight into whether behavior changes are sustained. For all of these reasons, we sought to quantify and compare the effects of the TGC preventive intervention on alcohol use frequency at the 12-month follow-up among youths with a CMC who reported high-risk alcohol use vs no or low-risk alcohol use at baseline, testing the hypothesis of no difference in effectiveness for these groups.

## Methods

### Study Design

This study was a prespecified secondary analysis of 12-month follow-up data from a single-center, 2-group, parallel randomized clinical trial. The trial was designed to examine the short-term and long-term effectiveness of the TGC psychoeducational intervention, which targets alcohol use and is tailored to adolescents with 3 types of CMCs. Study protocols for the original and extended follow-up and the statistical analysis plan are presented in Supplement 1. The Boston Children’s Hospital (BCH) Institutional Review Board reviewed and approved the study, with youth assent and a waiver of parental consent, as some youths may have chosen not to participate if parental consent was required, which would have biased the sample. All questionnaire data were collected via a secure electronic data capture tool and database, REDCap (Research Electronic Data Capture),^19,20^ hosted and managed independently at BCH. The study followed the Consolidated Standards of Reporting Trials (CONSORT) reporting guideline.

### Participants

**Figure 1.**
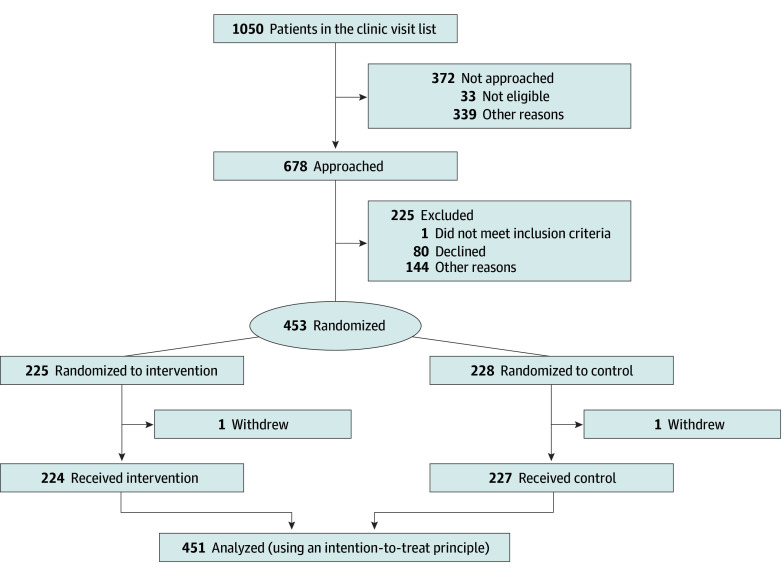
Study Flow Diagram

Between May 11, 2017, and November 20, 2018, patients aged 14 to 18 years seeking routine care in the endocrinology, rheumatology, or gastroenterology clinics at BCH were approached for study participation (Figure 1). Assessments were conducted at baseline, 6 months, and 12 months. Eligibility included having a clinical diagnosis (>1 year prior) of type 1 diabetes, juvenile idiopathic arthritis, systemic lupus erythematosus, or inflammatory bowel disease and being able to read English and use a tablet computer. Among the 678 adolescents approached, 451 (66.5%) participated in the baseline study (Figure 1).

### Randomization

Participants were approximately evenly assigned to the intervention or TAU using a stratified randomization scheme with block sizes of 2, 4, or 8, factoring in sex and age. The process was managed by a research statistician (L.E.W.).

### Follow-Up

Participants were contacted by telephone, text, or email for follow-up assessments at 6 and 12 months after the baseline assessment. Reminders for uncompleted surveys were sent; if needed, surveys were conducted over the telephone. Gift cards were sent upon survey completion. Participants turning 18 years of age during the follow-up were reconsented by telephone.

### Intervention

The TGC intervention^11^ included a deck of 28 to 32 slides that covered alcohol-related topics that had been identified in formative research as important to youths with a CMC and motivating for health-protecting decisions and behaviors.^10,21^ The slides featured visually appealing images created by an artist who worked in collaboration with the research team, with annotations drawn from formative research with youths with a CMC and subspecialty clinicians. Biomedical content within the TGC intervention was tailored to each disease area and addressed the specific effects of alcohol use on disease processes, treatment safety, and efficacy; psychosocial content was the same across disease areas and addressed personal identity, peer relations, and the importance of feeling well and in control of a chronic illness. All content was finalized with patient and clinician input, building from qualitative interviews^21^ (eMethods in Supplement 2), through an iterative process of review and revision.

The intervention was designed to be accessed on a tablet computer configured with a polarizing screen for privacy and self-review in the clinic waiting room. Participants could manually navigate through the slide deck or set the slides to autoplay, with an average viewing time of 4 minutes.

### Measures

#### Primary Outcome Measures

The primary outcome was self-reported frequency of alcohol use (in days) during the past 3 months, measured at baseline, 6 months, and 12 months using the following question: “In the last 3 months, on how many days did you have a drink containing alcohol?”^22^ Initiation of alcohol use was also evaluated at 6 and 12 months of follow-up.

The baseline high-risk alcohol use was defined as self-reporting of any alcohol-related injury, blackouts, emergency department (ED) visits, or vomiting in the past 12 months or heavy episodic or binge alcohol use in the past 3 months. The criteria for heavy episodic or binge use were defined as follows: female youths (aged 14-17 years) having 3 drinks containing alcohol on 1 occasion, female youths (aged 18 years) or male youths or youths of other genders (aged 14-15 years) having 4 drinks containing alcohol on 1 occasion, and male youths or youths of other genders (aged 16-18 years) having 5 drinks containing alcohol on 1 occasion. Participants who screened as negative for the past 12 months of alcohol use were assigned 0 and were included in the analysis.

Alcohol-related harms (blackout, injury, ED visit, or vomiting) were assessed with the following questions: “In the past 12 months, how often did you have blackouts while drinking alcohol when you couldn’t remember afterwards what had happened?”; “How often have you been injured during or after drinking alcohol?”; “How often have you gone to the emergency department because of problems related to your alcohol use?”; and “How often have you vomited or thrown up from alcohol use?” The responses (“never,” “once or twice,” “sometimes,” or “often”) were dichotomized as “any” vs “never” for the analysis.

#### Secondary and Exploratory Outcome Measures

The secondary outcome measures were alcohol health risk knowledge and alcohol risk intolerance. Alcohol health risk knowledge was defined as the percentage of correct responses for 7 or 8 questions about alcohol’s effects for the participant’s specific chronic illness (eTable 1 in Supplement 2). Higher scores indicated greater knowledge about alcohol health risks specific to the chronic condition. Alcohol risk intolerance was measured using a 6-point Likert scale to assess youths’ perceived riskiness of consuming different quantities of alcohol (eMethods in Supplement 2).

High-risk alcohol use behavior was defined as self-reporting any of the following at baseline: heavy episodic or binge drinking in the past 3 months or alcohol-related harms (blackout, injury, ED visit, or vomiting) in the past 12 months. The intervention effect on the score was modeled with mixed-effect models.

For the survey question “How many drinks on one occasion would you consider risky or dangerous drinking for yourself?” the 6-point Likert scale for the analysis was reverse coded such that a higher score indicated a higher intolerance of alcohol risk. For example, 5 indicates that any number of drinks per occasion is risky; 4, more than 1 drink per occasion is risky; 3, more than 2 drinks per occasion is risky; 2, more than 3 drinks per occasion is risky; 1, more than 4 drinks per occasion is risky; and 0, more than 5 drinks per occasion is risky/alcohol not risky. The intervention effect on the score was modeled with mixed-effect models. Exploratory outcomes included self-reported cannabis use in the past 3 months^22^ and nicotine use in the past 6 and 12 months.^23^

#### Baseline Measures

Self-reported demographic information was collected via a survey at baseline. Adolescents self-identified their gender (female, male, or another gender), and reported their school grade, highest level of parental education attained, race, and ethnicity. Race and ethnicity data were collected to account for variation in substance use behaviors by social group. Race was reported as Asian, Black, White, multiple races, or other race (including American Indian or Alaska Native, Native Hawaiian or Other Pacific Islander, and other race); ethnicity was reported as Hispanic or Latino. We measured adolescent high-risk alcohol use at baseline, defined as self-report of heavy episodic or binge drinking in the past 3 months or any alcohol-related harm adapted from the Personal Experience Screening Questionnaire^24^ in the past 12 months (eMethods in Supplement 2).

We inquired about heavy episodic or binge alcohol use using the following question: “In the past 3 months, have you had 3/4/5 or more drinks containing alcohol on 1 occasion?” Respondents answered “yes” or “no” (eMethods in Supplement 2).

### Statistical Analysis

Differential intervention effects over time were tested at 12 months for youths with a CMC who indicated no or low-risk alcohol use vs high-risk alcohol use at baseline, using an intention-to-treat principle. Under the assumption of data missing at random, we analyzed the full, incomplete dataset using maximum likelihood estimation. Generalized linear mixed-effects models with a negative binomial distribution and log link, along with a first-order autoregressive correlation structure, were used to test our hypothesis regarding differences in changes in alcohol use frequency during the past 3 months. This method uses each patient’s available data to compute maximum likelihood estimates, which estimate the parameter that is most likely to have resulted in the observed data.

An interaction term for time × group × risk status was used to assess differential intervention effects over time between participants with self-reported baseline no or low-risk alcohol use vs high-risk alcohol use. The adjusted models accounted for age group based on self-reported grade (middle or high school vs college or equivalent) and a proxy for socioeconomic status (highest level of parental education measured at baseline).

Among youths who reported no lifetime alcohol use at baseline, the intervention effects on initiation of alcohol use were assessed using logistic regression models. Youths with unknown alcohol status due to loss to follow-up or missing the alcohol use outcome at 6-month or 12-month follow-up were excluded from the analysis.

Sensitivity analyses for the alcohol use frequency outcomes were conducted by repeating the same adjusted models using 100 imputed datasets created by multiple imputation with the fully conditional specification regression and predictive mean matching method, assuming missing not at random. As exploratory analyses, the primary analysis was repeated, adjusting for baseline cannabis and nicotine use. Changes in the frequency of cannabis use and nicotine use were also assessed. Statistical significance was set at *P* < .05 (2-tailed). Data management and analyses were performed in SAS, version 9.4 (SAS Institute) and R, version 4.3.1 (R Project for Statistical Computing) between September 21, 2023, and February 3, 2024.

## Results

### Study Population

The randomized clinical trial included 451 participants with a mean (SD) age of 16.0 (1.4) years. There were 229 female youths (50.8%) and 217 male youths (48.1%); gender was unknown for 5 youths (1.1%). With regard to race, 8 youths (1.8%) were Asian, 18 (4.0%) were Black, 383 (84.9%) were White, 40 (8.9%) were of multiple races or other race, and 2 (0.4%) preferred not to answer. In terms of ethnicity, 39 youths (8.6%) were Hispanic or Latino, 408 (90.5%) were non-Hispanic or non-Latino, and 4 (0.9%) preferred not to answer.

Of the 451 participants, 212 (47.0%) were recruited from the diabetes clinic, 114 (25.3%) were recruited from the rheumatology clinic (for juvenile idiopathic arthritis or systemic lupus erythematosus), and 125 (27.7%) were recruited from the gastroenterology clinic (for ulcerative colitis or Crohn disease). Overall, 224 participants (49.7%) were randomized to the intervention group; the other 227 participants (50.3%) received TAU. The proportion of participants reporting parental educational attainment of a college degree was higher in the intervention group than in the TAU group (181 [80.8%] vs 162 [71.4%]) (Table 1). Otherwise, the treatment groups did not differ regarding sociodemographic factors. At the 12-month follow-up, 410 participants (90.9%) responded, including 404 (89.6%) who did not miss the questions related to alcohol use. Compared with those retained, the participants lost to follow-up were more likely to report experiencing alcohol-related harms and parents who did not complete college (eTable 2 in Supplement 2).

**Table 1.** Baseline Participant Characteristics^a^

Characteristic	All participants (N = 451)	Intervention group (n = 224)	TAU group (n = 227)
Age at baseline, mean (SD), y	16.0 (1.4)	16.0 (1.5)	16.1 (1.4)
Education level			
Middle or high school	387 (85.8)	193 (86.2)	194 (85.5)
College, vocational school, or other	64 (14.2)	31 (13.8)	33 (14.5)
Clinic			
Endocrinology	212 (47.0)	105 (46.9)	107 (47.1)
Rheumatology	114 (25.3)	57 (25.4)	57 (25.1)
Gastroenterology	125 (27.7)	62 (27.7)	63 (27.8)
Gender			
Female	229 (50.8)	115 (51.3)	114 (50.2)
Male	217 (48.1)	107 (47.8)	110 (48.5)
Other or unknown	5 (1.1)	2 (0.9)	3 (1.3)
Race			
Asian	8 (1.8)	2 (0.9)	6 (2.6)
Black	18 (4.0)	7 (3.1)	11 (4.8)
White	383 (84.9)	194 (86.6)	189 (83.3)
Multiple races or other race^b^	40 (8.9)	20 (8.9)	20 (8.8)
Prefer not to answer	2 (0.4)	1 (0.4)	1 (0.4)
Ethnicity			
Hispanic or Latino	39 (8.6)	18 (8.0)	21 (9.3)
Non-Hispanic or non-Latino	408 (90.5)	204 (91.1)	204 (89.9)
Prefer not to answer	4 (0.9)	2 (0.9)	2 (0.9)
Parental education level			
College or higher	343 (76.1)	181 (80.8)	162 (71.4)
Less than college	108 (23.9)	43 (19.2)	65 (28.6)
Alcohol use			
In past 12 mo, any^c^	108 (23.9)	60 (26.8)	48 (21.1)
In past 3 mo			
Any use	90 (20.0)	49 (21.9)	41 (18.1)
Frequency, mean (SD)^d^	0.9 (2.6)	1.0 (2.7)	0.8 (2.6)
Heavy episodic or binge drinking, any	39 (8.6)	20 (8.9)	19 (8.4)
Alcohol-related harms (blackout, injury, ED visit, or vomiting), any	33 (7.3)	18 (8.0)	15 (6.6)
High-risk alcohol behavior, any	52 (11.5)	26 (11.6)	26 (11.5)

Abbreviations: ED, emergency department; TAU, treatment as usual.

^a^ Unless indicated otherwise, values are presented as the No. (%) of participants.

^b^ Includes participants American Indian or Alaska Native, Native Hawaiian or Other Pacific Islander, multiple races, or other race.

^c^ Two participants did not contribute a response.

^d^ For the survey question “In the last 3 months, how many days did you have a drink containing alcohol?” responses from 447 participants were assessed (4 participants did not contribute).

### Primary Outcomes

**Figure 2.**
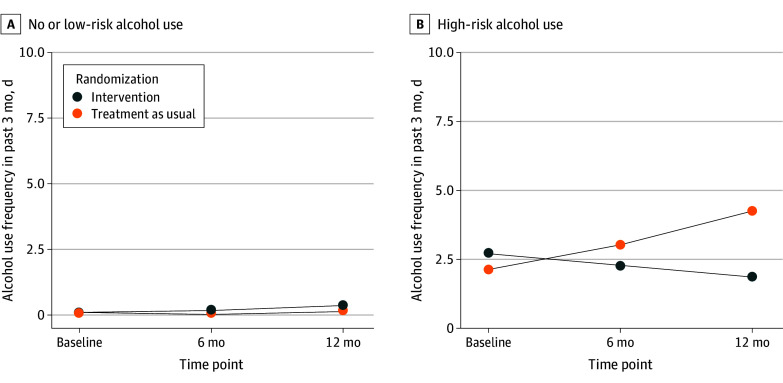
Intervention Effects on Alcohol Use Outcome, Stratified by High-Risk Alcohol Use Behavior at Baseline

Figure 2 and Table 2 depict the effects of the intervention on the primary outcome using interaction plots with marginal estimated values. Among the participants reporting high-risk alcohol use at baseline, the mean (SD) observed frequency of alcohol use during the past 3 months decreased from 6.3 (4.6) days at baseline to 4.9 (4.3) days at 12 months in the intervention group, whereas the frequency increased from 5.5 (4.9) days at baseline to 9.0 (5.8) days at 12 months in the TAU group (eTable 3 in Supplement 2). Among youths with a CMC reporting no or low-risk alcohol use at baseline, the mean (SD) observed alcohol use frequency increased from 0.3 (1.4) to 1.6 (3.8) days in the intervention group and from 0.2 (1.1) to 0.8 (2.3) days in the TAU group (eTable 3 in Supplement 2).

**Table 2.** Intervention Effects Stratified by High-Risk Alcohol Use Behavior at Baseline

Outcome (time × intervention)	Intervention effects (N = 451)^a^		*P* value for group interaction^c^
	ARRR or β regression coefficient (95% CI)^b^	*P* value	
Alcohol use frequency in past 3 mo			
High-risk use at baseline (n = 52)	0.60 (0.38 to 0.94)	.02	.02
No or low-risk use at baseline (n = 399)	1.17 (0.83 to 1.65)	.36	
Alcohol health risk knowledge^d^			
High-risk use at baseline	−2.95 (−10.35 to 4.46)	.44	.06
No or low-risk alcohol use at baseline	4.71 (2.16 to 7.26)	<.001	
Alcohol risk intolerance^e^			
High-risk use at baseline	0.12 (−0.33 to 0.58)	.59	.75
No or low-risk use at baseline	0.05 (−0.11 to 0.20)	.56	

Abbreviation: ARRR, adjusted relative rate ratio.

^a^ The intervention effect compares the difference in the changes in the past 3-month alcohol use frequency in days over time. All models included interaction between intervention and high-risk use vs no or low-risk use groups and were adjusted for the baseline demographics, including parental education level (less than college vs college or higher) and participant grade (middle or high school vs after high school [college or vocational school]).

^b^ For alcohol use frequency in past 3 months, ARRR values are reported. For alcohol health risk knowledge and alcohol risk intolerance, β regression coefficients are reported.

^c^ High-risk use vs no or low-risk use.

^d^ Alcohol health risk knowledge scaled score indicates the percentage of questions answered correctly out of 100. The β coefficients are shown as the intervention effect over time comparing the 2 groups (intervention and treatment as usual).

^e^ The β coefficients are shown as the intervention effect over time comparing the 2 groups (intervention and treatment as usual).

Among youths with a CMC reporting high-risk alcohol use at baseline, there was a significant time × intervention interaction, with a 40.0% decrease (adjusted relative rate ratio [ARRR], 0.60 [95% CI, 0.38 to 0.94]; *P* = .02) in the frequency of alcohol use over time for the intervention group relative to the TAU group. Among youths with a CMC reporting no or low-risk alcohol use, there were no group differences in changes in alcohol use frequency over time. Intervention effects differed between youths with high alcohol use and no or low-risk alcohol use (ARRR, 0.60 [95% CI, 0.38 to 0.94] vs 1.17 [0.83 to 1.65]; *P* = .02).

Sensitivity analysis with multiple imputations for missing data supported all findings with similar patterns of significance and effect size for youths with a CMC and with high-risk alcohol use and those with no or low-risk alcohol use at baseline (eTable 4 in Supplement 2). Among the 290 patients reporting no lifetime alcohol use, 47 (16.2%) reported initiation of alcohol use, with no group difference (eTable 5 in Supplement 2).

### Secondary Outcomes

**Figure 3.**
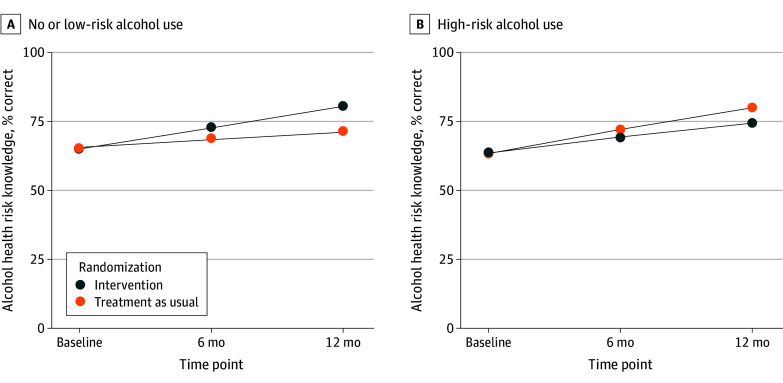
Intervention Effects on Alcohol Health Risk Knowledge, Stratified by High-Risk Alcohol Use Behavior at Baseline

Among youths with a CMC reporting baseline high-risk alcohol use, observed mean (SD) alcohol health risk knowledge (assessed as the percentage of correct responses) increased from 68.1% (22.2%) to 77.5% (23.4%) in the intervention group and from 65.2% (25.3%) to 82.2% (17.8%) in the TAU group (*P* = .06). Greater knowledge gains were found among youths with a CMC who reported no or low-risk alcohol use at baseline in the intervention group (mean [SD], from 63.7% [26.9%] to 79.3% [23.5%]) compared with the TAU group (mean [SD], from 64.6% [25.6%] to 70.5% [26.5%]) (β, 4.71 [95% CI, 2.16 to 7.26]; *P* < .001) (Figure 3, Table 2, and eTable 3 in Supplement 2).

There were no group differences in changes in alcohol risk intolerance regardless of reporting baseline high-risk alcohol use or no or low-risk alcohol use (Table 2). Inclusion of baseline measures of nicotine and cannabis use did not affect the overall results, nor were there effects on nicotine or cannabis use in exploratory analysis (eTables 6, 7, and 8 in Supplement 2).

## Discussion

In this secondary analysis, we found protective effects of a brief, self-administered preventive intervention for alcohol use tailored to the chronic illness experience at 12 months, suggesting that psychoeducational interventions that address youths’ health-related concerns and lived experiences can promote behavior change. This finding is consistent with prior reports.^25,26^ By observing participants over a relatively long period, we captured heterogeneities in and sustainment of intervention effects, including decreases in the frequency of alcohol use by treatment group among youths with a CMC who had already engaged in high-risk drinking at baseline and no change among youths with no or low-risk alcohol use.

Protective effects may derive from aligning the TGC model with theories of health behavior change. For example, these theories posit effects where interventions increase attention to a behavior, knowledge about its health effects, perceptions regarding the severity of harms that could result, perceived susceptibility to harms, and perceived benefits of avoiding the behavior.^27,28,29,30^ The TGC design features build from these theories and emphasize personalization to a youth’s specific chronic illness, with relatable and engaging visual, narrative, and medical content to convey the social-emotional meaning of alcohol use, risk of harms, and broad benefits of protecting health, identity, and symptom quiescence.^10,11,21^ An earlier evaluation of 6-month outcomes^11^ demonstrated favorable effects of the TGC intervention on knowledge and perceived riskiness of alcohol use; however, effects on these secondary outcomes were less clear for the longer-term risk-stratified outcomes. Knowledge scores increased in all groups over the 12-month period as participants learned more about alcohol through life experiences. Regardless of randomization status, participants with high-risk (vs low-risk) alcohol use may have learned about alcohol use from health care professionals, parents, or other adults in response to their alcohol consumption or harms, washing out any differences from the intervention. Although we were not able to detect knowledge differences between treatment groups among those reporting high-risk use, the intervention may have personalized knowledge to facilitate behavior change. For participants in lower-risk groups whose behaviors may not be eliciting information from outside influences, the intervention may be driving knowledge gains disproportionately resulting in the noted differences between the groups.

This research builds on reports about the efficacy of brief interventions for reducing substance use among adolescents and young adults,^31,32,33,34,35,36^ including a meta-analysis that found significant reductions in alcohol use with effects persisting for up to 1 year,^31^ extending the evidence base to medically vulnerable youth. The current study also adds to the growing body of evidence about the efficacy of electronic brief interventions for promoting health-protecting behaviors and reductions in substance use.^37,38^ Like other electronic brief interventions, TGC offers potential for fidelity, scale, and conservation of staff time.^39^ In our study, the intervention was delivered on a commercially available tablet in a clinic waiting room during a routine visit and thus did not require clinician administration or delivery. The intervention can be used remotely in alignment with the growth of telehealth for delivering clinical preventive services.^40,41,42,43,44,45^ Moving forward, it will be important to identify and evaluate pathways for sustainable implementation of TGC, for example, by testing acceptability and effects of delivery through a patient portal for home viewing. Although an electronic and self-administered approach may be especially appealing to youths who need or prefer flexibility in the tempo, timing, and setting for engaging with a brief intervention (factors that can affect acceptability and outcomes^43,45,46^), some youths may prefer or respond better to an in-person intervention; we do not know whether the effects would be similar or stronger if the approach were integrated into clinician workflows and messaging.

### Strengths and Limitations

The strengths of this study include the high retention of participants, use of structured measures, and inclusion of multiple chronic illness cohorts. Limitations include the low institutional and sociodemographic diversity. Most participants identified as White and reported having parents with a college education; although this aligns with the demographic profile of the study clinics, it may limit the generalizability of these findings. Future studies including youths with more diverse backgrounds are needed. Additionally, corrections for multiple comparisons were not applied in stratified analyses; thus, the results are exploratory. Replication studies are needed to test intervention effects in other settings and to adapt the intervention to other chronic illness conditions.

## Conclusions

In this secondary analysis of a randomized clinical trial of a brief chronic illness–tailored preventive intervention, alcohol use was effectively reduced among medically vulnerable youths at high risk of alcohol use and harm. This approach offers high potential for scale and merits future work to extend the intervention to address other chronic conditions and substances and to identify implementation pathways.

## Notes

Supplement 1.
Trial Protocol, Protocol Amendment Summary, and Statistical Analysis Plan

Supplement 2. **eTable 1.** Alcohol Health Risk Knowledge Survey Questions **eTable 2.** Baseline Characteristics by 12-Month Follow-Up Participation **eTable 3.** Observed Outcomes at All Assessment Occasions **eTable 4.** Sensitivity Analyses **eTable 5.** Intervention Effect on Alcohol Use Initiation Among Youths Reporting No Lifetime Alcohol Use at Baseline **eTable 6.** Cannabis and Nicotine Use at All Assessment Occasions **eTable 7.** Intervention Effects Stratified by Baseline High-Risk Alcohol Use Behavior, Adjusting for Cannabis and Nicotine Use **eTable 8.** Cannabis and Nicotine Use by Intervention Effects and High-Risk Alcohol Use Behavior at Baseline
eMethods

eReferences

Supplement 3.
Data Sharing Statement

## References

[1] Van Cleave J, Gortmaker SL, Perrin JM. Dynamics of obesity and chronic health conditions among children and youth. JAMA. 2010;303(7):623-630. doi:10.1001/jama.2010.104 20159870

[2] Nawi AM, Ismail R, Ibrahim F, . Risk and protective factors of drug abuse among adolescents: a systematic review. BMC Public Health. 2021;21(1):2088. doi:10.1186/s12889-021-11906-2 34774013 PMC8590764

[3] Kossowsky J, Weitzman ER. Instrumental substance use among youth with rheumatic disease—a biopsychosocial model. Rheum Dis Clin North Am. 2022;48(1):51-65. doi:10.1016/j.rdc.2021.08.003 34798959

[4] Kossowsky J, Magane KM, Levy S, Weitzman ER. Marijuana use to address symptoms and side effects by youth with chronic medical conditions. Pediatrics. 2021;147(3):e2020021352. doi:10.1542/peds.2020-021352 33542147

[5] Wisk LE, Weitzman ER. Substance use patterns through early adulthood: results for youth with and without chronic conditions. Am J Prev Med. 2016;51(1):33-45. doi:10.1016/j.amepre.2016.01.029 27039116 PMC4914415

[6] National Institute on Alcohol Abuse and Alcoholism. Get the facts about underage drinking. February 2024. Accessed February 22, 2024. https://www.niaaa.nih.gov/publications/brochures-and-fact-sheets/underage-drinking

[7] Weitzman ER, Ziemnik RE, Huang Q, Levy S. Alcohol and marijuana use and treatment nonadherence among medically vulnerable youth. Pediatrics. 2015;136(3):450-457. doi:10.1542/peds.2015-0722 26668849 PMC4552090

[8] Weitzman ER, Magane KM, Wisk LE, Allario J, Harstad E, Levy S. Alcohol use and alcohol-interactive medications among medically vulnerable youth. Pediatrics. 2018;142(4):e20174026. doi:10.1542/peds.2017-4026 30228168 PMC6317570

[9] Weitzman ER, Wisk LE, Levy S. Understanding the risk to medication adherence and safety of substance use behaviors for adolescents with chronic medical conditions: skipping, missing, and drug substitution behaviors. Int J Behav Med. 2016;23(1):S126.

[10] Wisk LE, Magane KM, Levy S, Weitzman ER. Alcohol use behaviors and reasons to abstain from or limit drinking among medically vulnerable youth. J Addict Med. 2020;14(4):311-318. doi:10.1097/ADM.0000000000000603 31985512 PMC7377949

[11] Weitzman ER, Wisk LE, Minegishi M, . Effects of a patient-centered intervention to reduce alcohol use among youth with chronic medical conditions. J Adolesc Health. 2022;71(4 suppl):S24-S33. doi:10.1016/j.jadohealth.2021.10.017 36122966

[12] Conrod PJ. Personality-targeted interventions for substance use and misuse. Curr Addict Rep. 2016;3(4):426-436. doi:10.1007/s40429-016-0127-6 27909645 PMC5110575

[13] Tinner L, Palmer JC, Lloyd EC, . Individual-, family- and school-based interventions to prevent multiple risk behaviours relating to alcohol, tobacco and drug use in young people aged 8-25 years: a systematic review and meta-analysis. BMC Public Health. 2022;22(1):1111. doi:10.1186/s12889-022-13072-5 35658920 PMC9165543

[14] Wisk LE, Magane KM, Nelson EB, Tsevat RK, Levy S, Weitzman ER. Psychoeducational messaging to reduce alcohol use for college students with type 1 diabetes: internet-delivered pilot trial. J Med Internet Res. 2021;23(9):e26418. doi:10.2196/26418 34591022 PMC8517820

[15] Gryczynski J, Carswell SB, O’Grady KE, Mitchell SG, Schwartz RP. Gender and ethnic differences in primary care patients’ response to computerized vs. in-person brief intervention for illicit drug misuse. J Subst Abuse Treat. 2018;84:50-56. doi:10.1016/j.jsat.2017.10.009 29195593 PMC5731246

[16] Gryczynski J, Mitchell SG, Gonzales A, . A randomized trial of computerized vs. in-person brief intervention for illicit drug use in primary care: outcomes through 12 months. J Subst Abuse Treat. 2015;50:3-10. doi:10.1016/j.jsat.2014.09.002 25282578 PMC4304885

[17] Levy S, Wisk LE, Minegishi M, . Association of screening and brief intervention with substance use in Massachusetts middle and high schools. JAMA Netw Open. 2022;5(8):e2226886. doi:10.1001/jamanetworkopen.2022.26886 35972741 PMC9382442

[18] Schulenberg JE, Maggs JL. A developmental perspective on alcohol use and heavy drinking during adolescence and the transition to young adulthood. J Stud Alcohol Suppl. 2002;63(14):54-70. doi:10.15288/jsas.2002.s14.54 12022730

[19] Harris PA, Taylor R, Minor BL, ; REDCap Consortium. The REDCap Consortium: building an international community of software platform partners. J Biomed Inform. 2019;95:103208. doi:10.1016/j.jbi.2019.103208 31078660 PMC7254481

[20] Harris PA, Taylor R, Thielke R, Payne J, Gonzalez N, Conde JG. Research electronic data capture (REDCap)—a metadata-driven methodology and workflow process for providing translational research informatics support. J Biomed Inform. 2009;42(2):377-381. doi:10.1016/j.jbi.2008.08.010 18929686 PMC2700030

[21] Weitzman ER, Salimian PK, Rabinow L, Levy S. Perspectives on substance use among youth with chronic medical conditions and implications for clinical guidance and prevention: a qualitative study. PLoS One. 2019;14(1):e0209963. doi:10.1371/journal.pone.0209963 30673730 PMC6343873

[22] Levy S, Wisk LE, Chadi N, Lunstead J, Shrier LA, Weitzman ER. Validation of a single question for the assessment of past three-month alcohol consumption among adolescents. Drug Alcohol Depend. 2021;228:109026. doi:10.1016/j.drugalcdep.2021.109026 34536715

[23] Levy S, Weitzman ER, Marin AC, Magane KM, Wisk LE, Shrier LA. Sensitivity and specificity of S2BI for identifying alcohol and cannabis use disorders among adolescents presenting for primary care. Subst Abus. 2021;42(3):388-395. doi:10.1080/08897077.2020.1803180 32814009

[24] Winters KC. Development of an adolescent alcohol and other drug abuse screening scale: Personal Experience Screening Questionnaire. Addict Behav. 1992;17(5):479-490. doi:10.1016/0306-4603(92)90008-J 1332434

[25] Jones A, Caes L, McMurtry CM, Eccleston C, Jordan A. Sociodevelopmental challenges faced by young people with chronic pain: a scoping review. J Pediatr Psychol. 2021;46(2):219-230. doi:10.1093/jpepsy/jsaa101 33211876

[26] Shorey S, Ng ED. The lived experiences of children and adolescents with non-communicable disease: a systematic review of qualitative studies. J Pediatr Nurs. 2020;51:75-84. doi:10.1016/j.pedn.2019.12.013 31926405

[27] Fisher JD, Fisher WA. Changing AIDS-risk behavior. Psychol Bull. 1992;111(3):455-474. doi:10.1037/0033-2909.111.3.455 1594721

[28] Harrison JA, Mullen PD, Green LW. A meta-analysis of studies of the health belief model with adults. Health Educ Res. 1992;7(1):107-116. doi:10.1093/her/7.1.107 10148735

[29] Bandura A. Self-efficacy: toward a unifying theory of behavioral change. Psychol Rev. 1977;84(2):191-215. doi:10.1037/0033-295X.84.2.191 847061

[30] Rosenstock IM, Strecher VJ, Becker MH. Social learning theory and the health belief model. Health Educ Q. 1988;15(2):175-183. doi:10.1177/109019818801500203 3378902

[31] Tanner-Smith EE, Lipsey MW. Brief alcohol interventions for adolescents and young adults: a systematic review and meta-analysis. J Subst Abuse Treat. 2015;51:1-18. doi:10.1016/j.jsat.2014.09.001 25300577 PMC4346408

[32] Steele DW, Becker SJ, Danko KJ, . Brief behavioral interventions for substance use in adolescents: a meta-analysis. Pediatrics. 2020;146(4):e20200351. doi:10.1542/peds.2020-0351 32928988

[33] Carney T, Myers B. Effectiveness of early interventions for substance-using adolescents: findings from a systematic review and meta-analysis. Subst Abuse Treat Prev Policy. 2012;7(1):25. doi:10.1186/1747-597X-7-25 22697269 PMC3538561

[34] Tait RJ, Hulse GK. A systematic review of the effectiveness of brief interventions with substance using adolescents by type of drug. Drug Alcohol Rev. 2003;22(3):337-346. doi:10.1080/0959523031000154481 15385228

[35] Halladay J, Scherer J, MacKillop J, . Brief interventions for cannabis use in emerging adults: a systematic review, meta-analysis, and evidence map. Drug Alcohol Depend. 2019;204:107565. doi:10.1016/j.drugalcdep.2019.107565 31751868

[36] Curry SJ, Krist AH, Owens DK, ; US Preventive Services Task Force. Screening and behavioral counseling interventions to reduce unhealthy alcohol use in adolescents and adults: US Preventive Services Task Force recommendation statement. JAMA. 2018;320(18):1899-1909. doi:10.1001/jama.2018.16789 30422199

[37] Doumas DM. Web-based personalized feedback: is this an appropriate approach for reducing drinking among high school students? J Subst Abuse Treat. 2015;50:76-80. doi:10.1016/j.jsat.2014.09.005 25448614

[38] Newton NC, Vogl LE, Teesson M, Andrews G. CLIMATE Schools: alcohol module—cross-validation of a school-based prevention programme for alcohol misuse. Aust N Z J Psychiatry. 2009;43(3):201-207. doi:10.1080/00048670802653364 19221908

[39] Gryczynski J, Mitchell SG, Schwartz RP, . Computer- vs. nurse practitioner-delivered brief intervention for adolescent marijuana, alcohol, and sex risk behaviors in school-based health centers. Drug Alcohol Depend. 2021;218(108423):108423. doi:10.1016/j.drugalcdep.2020.108423 33307377 PMC10329852

[40] Curfman AL, Hackell JM, Herendeen NE, ; Section on Telehealth Care, Committee on Practice and Ambulatory Medicine, Committee on Pediatric Workforce. Telehealth: improving access to and quality of pediatric health care. Pediatrics. 2021;148(3):e2021053129. doi:10.1542/peds.2021-053129 34462339 PMC9633975

[41] Wong CA, Madanay F, Ozer EM, . Digital health technology to enhance adolescent and young adult clinical preventive services: affordances and challenges. J Adolesc Health. 2020;67(2S):S24-S33. doi:10.1016/j.jadohealth.2019.10.018 32718511 PMC11925059

[42] Folk JB, Schiel MA, Oblath R, . The transition of academic mental health clinics to telehealth during the COVID-19 pandemic. J Am Acad Child Adolesc Psychiatry. 2022;61(2):277-290.e2. doi:10.1016/j.jaac.2021.06.003 34119633 PMC8607958

[43] Wood SM, White K, Peebles R, . Outcomes of a rapid adolescent telehealth scale-up during the COVID-19 pandemic. J Adolesc Health. 2020;67(2):172-178. doi:10.1016/j.jadohealth.2020.05.025 32611509 PMC7321038

[44] Barney A, Buckelew S, Mesheriakova V, Raymond-Flesch M. The COVID-19 pandemic and rapid implementation of adolescent and young adult telemedicine: challenges and opportunities for innovation. J Adolesc Health. 2020;67(2):164-171. doi:10.1016/j.jadohealth.2020.05.006 32410810 PMC7221366

[45] Li L, Childs AW. Using a patient safety/quality improvement model to assess telehealth for psychiatry and behavioral health services among special populations during COVID-19 and beyond. J Psychiatr Pract. 2021;27(4):245-253. doi:10.1097/PRA.0000000000000555 34398574 PMC8318385

[46] Cordova D, Alers-Rojas F, Lua FM, . The usability and acceptability of an adolescent mHealth HIV/STI and drug abuse preventive intervention in primary care. Behav Med. 2018;44(1):36-47. doi:10.1080/08964289.2016.1189396 27223646 PMC6201193

