# Supplementary material for: Disease-Tailored Brief Intervention for Alcohol Use Among Youths With Chronic Medical Conditions: A Secondary Analysis of a Randomized Clinical Trial
Source: JAMA Netw Open. 2024 Jul 10;7(7):e2419858. doi: 10.1001/jamanetworkopen.2024.19858 (PMC11238030; doi:10.1001/jamanetworkopen.2024.19858)
Supplement: Supplement 1. — Trial Protocol, Protocol Amendment Summary, and Statistical Analysis Plan [file jamanetwopen-e2419858-s001.pdf]

Children's Hospital Boston

IRB
Institutional Review Board

CHeRP
Children's Hospital eResearch Portal

CHeRP Protocol Outline:

TITLE: Trial of a novel brief intervention for substance use for youth with chronic medical conditions

A. Specific Aims/Objectives

The primary goal of this project is to develop a tailored screening, brief intervention and referral to treatment (SBIRT) model for youth with chronic medical conditions (YCMC) for delivery at point of care during a routine healthcare visit, through conducting a rigorous randomized control trial among adolescent patients with type-1 diabetes (T1D) or rheumatologic conditions. The secondary goal is to assess the attitudes and knowledge of parents of these same adolescents, to explore links between parent beliefs and adolescent behavior around substance use.

The need to develop accurate, feasible measures that inform screening, brief intervention, and referral to treatment (SBIRT) use, especially among medically vulnerable youth, is of great importance. Youth with chronic medical conditions are uniquely vulnerable to short term medical consequences from substance use. This group, that in total encompasses approximately 20-25% of US adolescents[1,2] is often overlooked. Large population-based studies of adolescent substance use do not capture information about medical conditions and disease registries do not capture substance use. As a result, little is known about this population and providers who care for these youth lack epidemiologically based protocols for screening and brief intervention among a medically vulnerable population of youth that very routinely accesses the healthcare system.

Seventy-five percent of the $2 trillion in US annual healthcare budget is spent on medical care of people with chronic illnesses,[3] of which $300 billion annually is attributable to treatment non-adherence.[4] Insofar as substance use constitutes a threat to an underlying chronic condition or adherence to self-care and treatment, secondary prevention built around screening and tailored/targeted advice in the medical home is among our most important, available, and underutilized interventions. This is especially so for youth where costs associated with chronic illness, substance use problems, and the negative interaction of the two compound over a lifetime. Where healthcare dollars are disappearing even as chronic, pediatric-onset medical and behavioral health problems are skyrocketing, it is imperative we advance knowledge-based models for secondary prevention.

The standard adolescent SBIRT model may not align with the specific needs and risks of the many youth growing up today with a chronic disease. Chronic diseases are heterogeneous in origin and mechanism but youth growing up with them who use substances experimentally or regularly are vulnerable to the potential for substances to negatively interact with: (a) their medications and treatment adherence, (b) lab tests used to diagnoses and track their condition, (c) self-care and disease management regimens. Additionally, substance use can expose YCMC to behaviors and lifestyle factors that may acutely jeopardize their health and contribute to disease activity, including: poor/insufficient sleep, deviation from a dietary program or nutrition plan, secondhand smoke exposure in parties and pub settings, and unplanned/unprotected sex with attendant risks from STDs and pregnancy—risks that may be life threatening for youth taking immune-suppressing or teratogenic medications. These risks can affect all youth but they pose especially acute dangers for YCMC. For all of these reasons, **standard screening thresholds for assigning youth to "risk groups", triggering interventions and cuing guidance which are based on age and frequency of use may differ for medically vulnerable versus healthy youth**. Screening thresholds developed with healthy youth may underestimate true risk for YCMC; guidance that does not reflect the chronic disease context may "miss the mark" by ignoring mention of near term dangers and behavioral motivators.

Parents are key stakeholders in addressing adolescent substance use. For example, two-thirds (66%) of adolescent patients surveyed in our NIAAA study of alcohol use risk among medically vulnerable youth rate “not wanting to disappoint their parents” as a “very important” reason for choosing to limit their alcohol use, when they do use. Nevertheless the standard model for adolescent SBIRT does not include parents as recipients of parallel messaging and psycho-education. Because parents’ beliefs regarding the safety and normalcy of AOD use for their children are largely unexplored there is limited potential for addressing them in adolescent SBIRT, or for leveraging parental concerns and actions as part of SBIRT messaging and psycho-education. In the best case, parents are assumed to hold values and beliefs supportive of delayed onset and non-use of substances by their children, reinforcing SBIRT recommendations. In reality, parents may interpret their child’s use of substances in social situations as an unavoidable “developmental stage” or even a prized sign of normalcy, inclusion and social success. This may be especially so for the many youth growing up with a chronic disease. Mixed messages, tacit or even explicit approval from parents may exacerbate a child’s risk. In the case of chronically ill youth, substance use may also reflect attempts to mitigate symptoms or side effects as when marijuana is used to treat pain or nausea, possibly with a parent’s support, which can muddy prevention messages delivered to youth

This project is the second component of a two part study in which we will enroll a total of 900 adolescents as indicated by Figure 1. In this project, we will create a tailored SBIRT protocol that reflects YCMC specific concerns and risk thresholds. We will include YCMC in the general longitudinal cohort study being undertaken in the Adolescent/Young Adult Medicine clinic, and additionally enroll these youth into an embedded trial of a tailored SBIRT intervention in which we will assess acceptability and effects of the tailored SBIRT model. **For this protocol, we will be discussing the “Youth with Chronic Medical Conditions” cohort. We will enroll a total of 450 participants from the Rheumatology and Endocrinology Clinics (225 participants from each) for this study and will attempt to follow participants for one year. Accounting for attrition at 6 months and 12 months follow-up, we expect 300 participants will complete the entire study (see figure 1 for projected enrollment numbers).**

We will recruit a convenience sample of adolescent patients (n=450) ages 14-17 years presenting for routine specialty care at Boston Children’s Hospital (BCH) Rheumatology and Endocrinology clinics. Eligible and consented participants will be randomized into intervention or control arms of the trial. Participants will complete a baseline assessment at the time of enrollment and then follow- up assessments at 6 and 12 months after the baseline assessment. Each assessment battery will include 1) questions about frequency of use, 2) candidates of intermediary measures that may predict future use (for example, reports about perceived harm of use), 3) substance specific measures of harms associated of use, 4) disease specific measures such as pain interference and emergency care received to assess disease burden and disease management and 5) Time Line Follow Back Calendar as criterion standard measures of substance use frequency. Those in the intervention arm will receive a computerized brief intervention composed of tailored feedback and psycho-education specific to their chronic condition. Participants in the control arm will receive brief advice on the unrelated topic of internet safety. We hypothesize that YCMC who receive the intervention will be less likely than their peers to initiate or escalate alcohol consumption 6- and 12-months post intervention. And, YCMC who receive the intervention will be less likely than their peers to initiate or escalate marijuana use 6- and 12-months post intervention.

We will recruit in tandem at baseline only, parents of those youth who are enrolled in the study, to briefly assess their attitudes, knowledge and beliefs around substance use and YCMC, as well as their opinions on various risk behaviors and health concerns. We will explore whether parent beliefs at baseline have any correlation with their child’s substance use behavior and/or attitudes and perceptions of risk.

Figure 1: Study Enrollment Plan

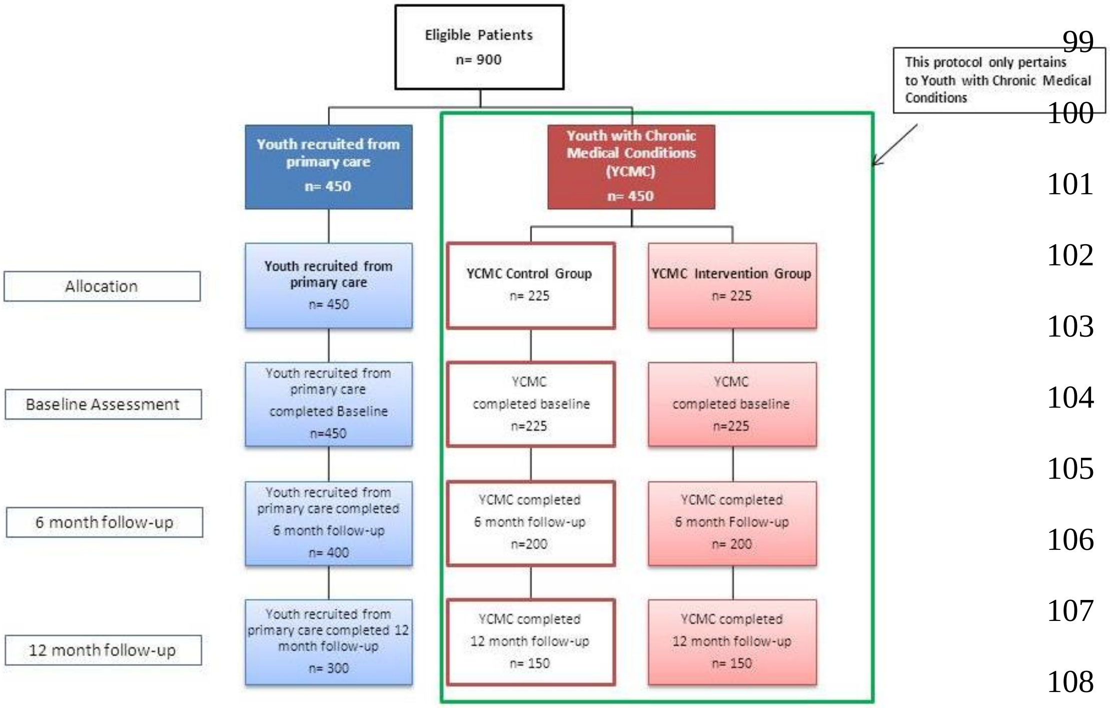

B. Background and Significance

The outstanding unmet need this project seeks to address is the need to develop accurate, feasible measures that inform SBIRT use among medically vulnerable youth. YCMC are uniquely vulnerable to short term medical consequences from substance use. Large population-based studies of adolescent substance use do not capture information about medical conditions and disease registries do not capture substance use. As a result, little is known about this population and providers who care for these youth lack epidemiologically based protocols for SBIRT among a medically vulnerable population of youth that very routinely accesses the healthcare system.

1. Risk of substance use during adolescence. The developing adolescent brain is particularly vulnerable to the toxic effects of alcohol, marijuana, and other drug use. In addition to acute consequences, early initiation of substance use is associated with increased odds of developing a substance use disorder (SUD) and experiencing substance-related problems, even as an adult. [5,6] Compared to those who delay initiation of alcohol use into young adulthood, those who begin to drink during early adolescence are five times more likely to develop alcohol dependence or abuse[6,7] and four times more likely to develop marijuana addiction.[8] Adolescents who use alcohol are at increased risk of alcohol-related accidents and injury rates even if they do not develop addiction.[9] Marijuana use during adolescence is associated with decreases in neurocognitive functioning.[10] Particularly for adolescents, no amount of alcohol or drug use is "safe"; harms from substance use accrue at all levels of consumption. In fact, at a population level, morbidity and mortality burdens are driven by the non-zero risk for harm derived from use by the majority.[11] Thus even interventions with small impacts at the individual level can result in large public health benefit.

2. Enabling the study of alcohol and marijuana screening and brief intervention. Screening and brief intervention is effective and efficient for reducing alcohol consumption[12–15] and overall health care costs[16,17] in adults and is recommended for adolescents,[15,18] though the literature has not firmly established the most effective approaches. The American Academy of Pediatrics (AAP), [15] other

professional societies, and multiple governmental institutions[19–22] have identified and invested in brief interventions as the most promising approach to addressing adolescent substance use because of their low cost and few, if any, risks. A review by the U. S. Preventive Services Task Force found the research base insufficient to evaluate the effectiveness of brief interventions for substance use among adolescents and identified a need for rigorous trials.[23] The opportunity to develop and refine outcomes measures provides a unique opportunity to prepare the field for advancing this gap.

3. Detecting small changes in behavior. Even small reductions in substance use may have important public health benefit, but are difficult to detect in small samples over short timelines. Efforts to advance adolescent SBIRT in primary care settings aim to achieve reductions in substance use and consequent harms. The nascent literature suggests that brief interventions for adolescents are most promising when targeted at primary and secondary intervention to delay initiation among those that have not used yet and to decrease use among those that have.[24] Typical individual outcomes could include, for example, a 6-month delay in onset of alcohol use or prevention of the escalation of marijuana use often seen in the high school years. On a population level, the accrual of small impacts across large cohorts can have a major cumulative effect on improving public health. [11] However, detecting small incremental changes arising from interventions in order to establish effectiveness is practically and scientifically challenging. Comprehensive assessment approaches are lengthy and burdensome to administer which precludes their use in pragmatic trials and clinical settings. Consequently, we are unable to capture from large, real life health care settings those data needed to assess impacts.

4. Tailoring and testing an adolescent SBIRT model for YCMC. Youth growing up with a chronic disease who use substances experimentally or regularly are vulnerable to the potential for substances to negatively interact with: their medications and treatment adherence, lab tests used to diagnose and track their condition, and self-care and disease management by regimens. Additionally, substance use can expose YCMC to behaviors and lifestyle factors that may acutely jeopardize their health and contribute to disease activity, including: poor/insufficient sleep, deviation from a dietary program or nutrition plan, secondhand smoke exposure in parties and pub settings, and unplanned/unprotected sex with attendant risks from STDs and pregnancy—risks that may be life threatening for youth taking immune-suppressing or teratogenic medications. These risks can affect all youth but they pose especially acute dangers for YCMC. For all of these reasons, standard screening thresholds for assigning youth to “risk groups”, triggering interventions and cuing guidance which are based on age and frequency of use may differ for medically vulnerable versus healthy youth. Screening thresholds developed with healthy youth may underestimate true risk for YCMC; guidance that does not reflect the chronic disease context may “miss the mark” by ignoring mention of near term dangers and behavioral motivators.

5. Developing and testing the efficacy of a brief electronic intervention. A model of delivering screening, tailored feedback and psycho-education electronically and complemented by physician advice at the point of care takes advantage of the migration to electronic clinical infrastructure of data captured at the point-of-care and the use of tablets and apps to collect patient-reported outcomes to augment healthcare decision-making. Point-of-care data capture enables analyses of clinical data from large numbers of adolescents seen in practices where this SBIRT model is used, informing understanding of impacts of interventions delivered in real world settings. This model of screening and intervention delivery is efficient insofar as it reduces burdens on physician time and training-providers do not have to elicit information for patients and the informational portion of the intervention is delivered electronically. The model builds on an approach with proven efficacy for reducing alcohol initiation among healthy youth in a primary care setting. [24]

C. Preliminary Studies

Validation of screening in youth with Chronic Medical Conditions (YCMC) Drs. Levy and Weitzman are currently co-principal investigators (PIs) on an NIAAA funded study Validating NIAAA's Brief Screening Guide in Youth with Chronic Medical Conditions (R01-AA021913). This screening validation study is exploring alcohol use risk among youth with chronic medical conditions (YCMC) ages 9-18 years (n=400) receiving care at BCH. Among 403 who have completed baseline assessments, past year alcohol use was reported by nearly one third (30.8%) of the entire sample, and over one third (36.5%) of high school youth. Binge drinking was reported by 10.4% of the total sample, by 12.7% of high school youth, and 37.7% of high school youth who reported past-year alcohol use. Approximately one sixth of the entire sample (17.2%) and one fifth (20.6%) of high school youth reported using marijuana in the past year. Sixty percent report being asked in the past year by a member of their care team about their drinking.

Screening rates and practices among physicians. Levy collaborated with the Massachusetts Department of Public Health on a study to determine alcohol and drug screening rates and practices among physicians practicing in Massachusetts. A large majority of respondents (86%) reported that they screen adolescents for substance use annually though only a minority (34%) reported using appropriately validated tools, leading to the potential for significant under appreciation of substance use problems and disorders.[25] An outcome of this project was the creation and publication of a clinical practice guideline for adolescent screening, brief intervention, and referral to treatment (SBIRT) that was described as "easy to understand and use" during field testing, and ultimately distributed to every practicing pediatrician and family physician in the state.[20] An updated version of the guideline was subsequently included in a published policy statement by the American Academy of Pediatrics[14].

Screening, Brief Intervention, Referral to Treatment (SBIRT) curriculum development. Levy is PI on a Substance Abuse and Mental Health Services Administration (SAMHSA)-funded medical residency SBIRT curriculum development project (TI020267). The team designed and adapted SBIRT strategies for various areas in the hospital, including General Adolescent Medicine, Psychiatry, Developmental Medicine and the Emergency Department (ED), and then trained residents as they rotated through each area. After the second year of curriculum implementation, trainee satisfaction is very high with over 90% agreeing that materials have enhanced their skills, are useful and applicable. Mean post-training knowledge assessments ranged from 72-89% and post-visit forms documented appropriate screening and brief intervention steps in 71-100% of instances. These results were presented at the 2011 Association for Medical Education and Research in Substance Abuse [15] national meeting (manuscript in preparation).

ED SBIRT tool. Levy developed an electronic SBIRT screening algorithm that is currently used in BCH ED. Resident physicians explain confidentiality to parents and patients aged 12-18 and then use the ED SBIRT tool to determine substance use risk categories with an electronic tool that uses branching logic. A summary sheet with the adolescent's risk category and recommended physician intervention automatically prints upon completion, enabling residents to counsel patients about substance use or seek support from an ED mental health worker as necessary, as per tool guidelines. Formative evaluation indicates high acceptability, utility and minimal problems in the 2 years of operation.

Engaging chronically ill youth with self-care and health information. Weitzman is testing models for engaging chronically ill youth with patient-centered health information technology (HIT) to support improved activation in self-care, communication with a care team, monitoring and reporting of behavioral health issues related to disease management, outcomes and successful transition to internal medicine.[16] She leads multiple investigations into beliefs, perceptions and readiness for health care transitions (HCT) among YCMC, including in relation to health risk behaviors.[17] Adolescents generally report high levels of willingness to report and share health data,[18,19] though new work also finds some reticence to share alcohol data. Surveying repeat users of the BCH personal health record (PHR) system (total N=261, response rate 56%), she found that 15% of patients/parents would be *reticent* to share youth alcohol use data stored in their PHR with a provider outside of BCH in order to support care improvement. Odds of reticence were almost twice as high for information concerning alcohol use than for contagious illness (adjusted OR 1.8, 95% CI 1.2, 2.6) due to concern for

relevance (52%), disclosure to insurance (47.6%) and/or family (20.5%). Reticence to share alcohol use data across settings underscores the importance of screening at the point of care to ensure data are available.[20]

Alcohol Use among YCMC and Medication Adherence. Weitzman surveyed chronically ill patients ages 16-25 from the BCH Diabetes, Cystic Fibrosis, Myelodysplasia, and Adolescent Medicine clinics (n=78, response rate 34%). Reports of past-30 day alcohol use include: 56% any alcohol, 27% drank 6+ times, 34% binged 1+ time. Patients who reported any past 30 day alcohol use had higher levels of improper medication use than patients who reported no alcohol use (OR 4.3, 95% CI 1.02, 18.4, p=.04). Frequencies of past 30 day drinking and medication non-adherence were correlated (correlation coefficients .34-.48, all p-values <.05).

Developing electronic surveys, assessments and tools. Our team has considerable experience with electronic assessment and survey research. Levy has implemented an SBIRT screening algorithm electronically for use in the BCH ED and Weitzman has considerable experience with electronic surveys of varying levels of complexity including those that provide access to contextualized personal health information and geographical information systems (GIS) displays of biosurveillance data.[21–23]

D. Design and Methods

Study Design

The proposed project is a randomized control trial to assess the efficacy of an SBIRT model tailored to youth with chronic medical conditions. This trial is being built into a larger, longitudinal cohort study to assess whether a set of brief, substance specific questions can accurately predict adolescent substance use outcomes when compared to more lengthy, criterion standard assessment questions, in cross-sectional and prospective analysis. The first component of the project will consist of pre-testing an assessment battery and brief electronic intervention with a small sample of 14-17 year old youth. In the second component we will randomize consented eligible participants into intervention or control arms of the study and administer the 1) Baseline Assessment Battery and the 2) Brief Intervention OR Brief Control Information. In the third component we will follow up with all participants at 6 and then 12 months after study entry to reassess rates of substance use through the follow up assessment batteries.

At baseline, we will also attempt to enroll parents of all participants who are present at the time of enrollment. We will separately consent and administer a brief assessment battery to parents to assess their knowledge and attitudes of YCMC substance use, as well as opinions and attitudes regarding other health and risk behaviors.

Patient Selection and Inclusion/Exclusion Criteria

Inclusion criteria: 14-17 year-old youth presenting for routine medical care in the Rheumatology clinic or Endocrinology clinic at BCH, with informed assent. To be eligible, participants in the Endocrinology clinic must have a diagnosis of type 1 diabetes for at least a year and participants in the Rheumatology clinic must have a diagnosis of a rheumatologic condition for at least a year. Eligible youth must also be between 14-17 years old at the time of enrollment and be able to read and understand English at a middle school level or greater. Participants must consent to participation in the study and consent to the 6 month and 12 month follow up assessments.

Parents whose children enrolled in the study, and who are able to read and understand English at a middle school level or greater will be eligible to participate in the parent study.

Exclusion criteria: Patients who are medically or emotionally unstable or otherwise unable to provide assent at the time of their appointment as determined by their clinician or the research team, those who are unable to speak/read English at a middle school reading level, use a computer keyboard and/or complete an interviewer-assisted questionnaire will be excluded. Patients who do not consent to 6 month and 12 month re-assessment will also be excluded. Patients who are pregnant at baseline

will be excluded from the study. If a participant enrolls in the study and becomes pregnant after baseline, they will not be excluded from the follow up assessments.

Parents of enrolled participants who are not able to read and understand English at a middle school level or greater will be excluded. Parent ineligibility will not affect patient eligibility.

Recruitment

At baseline, a project Research Assistant (RA) will screen the Rheumatology and Endocrinology Clinic rosters (through PowerChart, Hyperspace, and/or Childrens360) to generate a roster of potentially eligible patients with upcoming outpatient appointments, including the Center for Ambulatory Transfusion (CAT-CR).The roster will be given to clinicians or the administrator within the department for approval if requested. The providers will review the list and let the RA(s) know which patients are okay to approach to invite to participate in the study.

The RA(s) will use the clinic roster to contact these eligible patients prior to their clinic appointment. The RA will approach participants at the time of their appointment to ask about their interest in participating in a research project. The RA will meet with interested and/or eligible patients in a private space in or near the clinic or the clinic waiting room to explain the purpose and details of the study to interested patients, and answer any questions. For all those who are interested in participation, the RA will verbally verify if the participant is able to read English at a middle school level or higher and confirm that the patient is eligible to participate in the study. For those who are eligible to participate in the study, the RA will then obtain assent and the participant will be randomized into either the intervention or control group. The RA will then administer the recruitment form, measurement battery, and either the test intervention or control intervention based on randomization. At baseline, participants will be consented for the baseline assessment battery, 6 month follow up, and 12 month follow-up. If the patient is interested in participating but cannot stay to complete the assessment battery, the RA will ask the patient if they would like to participate at their next scheduled clinic appointment of if they are interested in coming in at a scheduled time to complete the study. The RA will collect multiple forms of contact for each participant at baseline enrollment and will ask the participant to indicate which method(s) are preferred for re-contact at 6 and 12 months post baseline.

The primary aim of this study is to develop a tailored screening, brief intervention and referral to treatment (SBIRT) model for youth with chronic medical conditions (YCMC) for delivery at point of care during a routine healthcare visit, through conducting a rigorous randomized control trial among adolescent patients with type-1 diabetes (T1D) or rheumatologic conditions. Adolescents will be invited to participate in the study as per the primary goal of the project. If their parent is present at the time of recruitment, the parent will be co-informed of the study and the adolescent is encouraged to speak to their parent before deciding to be part of the study. After the adolescent is consented into the study, if his/her parent is also present and co-informed of the study, we may approach the parent to invite them to participate in a survey for the secondary goal of this project. The secondary goal of this project is to assess the attitudes and knowledge of parents of these same adolescents, to explore links between parent beliefs and adolescent behavior around substance use. Parental participation is not a requirement for the child's participation. If a child comes to the clinic and their parent is not present the child is still invited to participate in the study and encouraged to talk to their parent before and after they decide to participate in the study. However these parents will not be approached at a later time to participate in the research study because they were not co-informed of the study by the RA.

The RA will explain to the parent the purpose and details of the second aim of the study and answer any questions. If the parent is interested in participating, the RA will verbally confirm that the parent is able to read English at a middle school level or higher and will then consent and enroll the parent.

The RA will administer a brief assessment battery to the parent. The parent's study ID number will be linked to their child's ID number at baseline. No follow up will occur with enrolled parents. Parents will not be re-contacted for this study after baseline participation.

For re-administration of the adolescent assessment battery, an e-mail will be sent to all participants with a link to a 6 month follow up survey and another e-mail with a link to a 12 month follow up survey. In order to maximize follow up rates, the recruitment window for Research Assistants to contact patients will be 5-8 months post baseline for the 6 month follow up battery and 11-14 months post baseline for the 12 month follow up battery. An e-mail will be sent to participants with a link to the follow up survey. E-mail reminders will also be sent to those that receive the survey through e-mail but do not complete them. If for some reason completing the follow-ups through e-mail is not possible or participants do not complete them after receiving the reminders, the survey can be administered over the phone by one of the research staff.

At the 6-month and 12-month follow up, each participant will be sent an email that reminds them about the study, and explains the purpose and details of the follow up assessment. Each email will contain a link to the follow-up survey that is attached to the individual participant's study ID number and administered through REDCap. Surveys sent to participants will follow all privacy and confidentiality guidelines as set through REDCap and BCH. Participants will receive two reminder emails to complete the follow-up assessment surveys online. If a participant has not completed the follow-up assessments after two reminders, the RA will attempt to contact the participant by phone and/or text to remind them about the follow up and request completion, or to administer the follow-up assessment by phone if the participant is unable to complete the battery online. Before administering the assessment battery by phone, the RA will ask the participant to verify that he/she is in a private setting where their answers cannot be overheard by others.

Participants who have turned 18 by the time of follow up will be re-consented over the phone prior to completing the follow up surveys through e-mail. The RA will administer the consent form over the phone and obtain verbal consent in lieu of the written assent form administered at baseline

Upon completion of the survey online, the RA will receive a notification from REDCap that the survey has been completed, and will send the participant remuneration, either in the form of electronic gift card via email, or physical gift card via mail, depending on participant preference.

Description of Study Treatments or Exposures/Predictors

This is a randomized control trial. We are interested in seeing if a brief electronic intervention that provides disease-specific information related to substance use as well as psycho-education on physical and mental well-being will have an impact on future substance use behaviors, knowledge, and perceptions.

Definition of Primary and Secondary Outcomes/Endpoints

The primary goal of the proposed project is to develop an adolescent SBIRT model tailored to YCMC and to evaluate its acceptability and effects using a randomized-controlled trial. Building onto an earlier phase of this project (Validating Adolescent SBIRT Measures), we will assess the validity and acceptability of a subset of brief substance-specific questions that are strongly associated with reports of current substance use behaviors and harms and that predict future substance use behaviors and harms. Screening, tailored feedback and psycho-education will all be delivered electronically. The brief intervention will target perceived risk of harm of a given substance, knowledge about substances, knowledge of disease specific and medication interactions, and intentions to use. These intermediary measures may serve as proxy markers that can be tracked and may predict substance

use trajectories during a long term follow up period.

We hypothesize that YCMC who receive the intervention will be less likely than their peers to initiate or escalate alcohol consumption 6- and 12-months post intervention. And, YCMC who receive the intervention will be less likely than their peers to initiate or escalate marijuana use 6- and 12-months post intervention.

Data Collection Methods, Assessments, and Schedule (what assessments performed, how often)

Pilot Testing: We have developed the psycho-educational intervention which includes information on substance use and chronic disease knowledge and brief advice as well as a control "intervention" on internet safety. We will pre-test the baseline assessment battery and the brief interventions with 10 youth from the BCH Endocrinology and Rheumatology clinics, as well as the CATCR. We will invite youth aged 14-17 to meet individually with RAs who will show them the substance use intervention or the control intervention and the baseline assessment battery. The RA will ask participants to rate overall understandability and word choice of the intervention and the questionnaires. The RAs will also demonstrate and allow participants to click through the intervention and assessment battery and ask them to comment on the design, ease of use, helpfulness of the directions, and any areas of confusion. The RAs will record participant responses and compare notes following the interview. The assessment battery and intervention will be adapted as needed based on participant comments.

Assessment Battery: The baseline assessment battery will be completed before administering the intervention/control. Participants in both the intervention and control arms will complete the baseline, 6 month, and 12 month assessment batteries. Our assessment battery will consist of 1) health information including medical history 2) factors that may affect substance use such as social support, family structure, extracurricular activities, and mental health 3) measures for alcohol, tobacco, marijuana, prescription drug, and other illicit drug use frequency and severity; which will include questions about the participant's own use and friend's use 4) intermediary measures related to substance use such as perceived attitudes, knowledge, fears, availability, and future use will be included as well as measures related to the participant's personality such as self-efficacy and grit 5) disease specific questions related to burden of disease and disease management. When possible, we will use previously validated tools selected for high clinical and research utility, parsimony, ease of administration and interpretations. At baseline, questionnaire responses will be entered into the tablet computer by the participant. During the 6 month and 12 month follow up, assessment batteries will be administered over the phone or in person by the RA.

The assessment battery consists of questions from previously validated surveys, as well as novel questions that were created for this study. The goal was to develop an assessment battery that was easy to administer, brief, and had good predictive validity for youth. In instances where we wished to calculate the total score for each participant such as the 8-Item Grit Scale[36] and the Patient Health Questionnaire-2 (PHQ-2)[37], the entire questionnaire was included. For measures where we were interested in a more specific outcome such as grades in school or injuries related to alcohol, we selected questions from larger scales such as the Monitoring the Future study or the Personal Experience Screening Questionnaire (PESQ)[38] based on face validity or psychometric properties (when available). Assessment Battery measures include the following domains as outlined in the table below. Please see appendix for a complete copy of the assessment battery.

Question #s	Measure/ Domain	Name of tool(s)
**Eligibility and Demographics**		

	Recruitment Form	
1-3	Participant Eligibility	
4-6	Demographics – age, grade, gender	
**Health Information and other predictors of substance use**		
7	Overall health	Patient Reported Outcomes Measurement Information Systems (PROMIS) Adult Short Form Survey (V1.1 Global Health) [39]
8-9, 18-23	Diabetes survey only – frequency of severe hypo/hyper glycemic episodes medical history related to diabetes	• Novel
10	Rheumatology survey only – Frequency of disease flare	• Novel
11-13 16-17	Medical and psychiatric history	• National Survey on Children's Health (NSCH)[40]<br>• Novel
24	Stress related to diabetes	• Problem areas in Diabetes – Teen Survey[41]
14-15	Family medical history	Novel
57	Sleep	Youth Risk Behavior Survey (YRBS) [42]
58, 73-74	Social support	• Parental Monitoring and Communication Scale[43]<br>• PROMIS Adult Short Form Survey v2.0 Emotional Support 6a, Social Support for Adolescents Scale[44]
106-107 (4 items)	Current Mental Health Status	• Patient Health Questionnaire (PHQ-2)[45]<br>• Generalized Anxiety Disorder scale (GAD-2)[46]
**Alcohol, Tobacco, Marijuana, and use of Other Substances**		
25-35	Prescription medication use	• NSCH[40]<br>• Novel adapted from McCabe SE, et al. 2007[47]<br>• Developed for *Validating NIAAA's Brief Screening Guide in Youth with Chronic Medical Conditions* (R01-AA021913)
36-43	Substance Use Screen	Screening to Brief Intervention (S2BI) [48]
44-45, 47, 48-49	Criterion standard for substance use disorder (SUD) diagnosis: Lifetime reported use and past 30 day reported use	• Computerized Diagnostic DISC-IV[30]<br>Monitoring the Future (MTF) [49]
46	Use of marijuana as medicine	Novel
50, 55-56, 53, 75, 80, 85	Brief substance consumption frequency measures	Developed for *Validating NIAAA's Brief Screening Guide in Youth with Chronic Medical Conditions* (R01-AA021913)
51-54	Alcohol Safety Screen	Developed for *Validating NIAAA's*

		Brief Screening Guide in Youth with Chronic Medical Conditions
	Criterion standard substance use frequency measure	90-day Timeline Follow Back Calendar[50]
**Intermediary Measures (Attitudes, Knowledge, and Alternatives)**		
59-62, 64	Time spent with alternative activities	• Novel<br>• YRBS[42]
63 (3 items)	Perception of overscheduling	Measure of Perceived Over-Scheduling (MOPS)[51]
65 (4 items)	Acceptability and interest in app/tools to monitor health behaviors	• Novel
66-68	Internet and social media use	• Novel
69-72	Academic and extracurricular performance	• CASA Columbia[52]<br>• Novel
86 (14 items), 88	Possible reasons to abstain from alcohol and marijuana use	Developed for *Validating NIAAA's Brief Screening Guide in Youth with Chronic Medical Conditions*
87	Confidence in refusal skills	Novel
95 (9 items)	Knowledge about the impact of marijuana and alcohol on health.	Novel
96 (9 items), 97	Perceived risk of harm from substance use	MTF [49]
98-100	Perceived availability of alcohol and marijuana	MTF[49]
101 (4 items)	Perceived likelihood of future substance use	MTF[49]
102-105	Receipt of healthcare advice about substances from a medical professional	Developed for *Validating NIAAA's Brief Screening Guide in Youth with Chronic Medical Conditions*
108 (8 items)	Grit: Perseverance and passion for long term goals	8-Item Grit Scale[36]
109	Future goals for after high school	Novel
**Substance Specific Patient Centered Outcomes**		
76-79, 81-84	Experienced problems and consequences associated with alcohol and marijuana use	• CIDI-SAM[53]<br>• Novel<br>• Personal Experience Screening Questionnaire (PESQ)[38]<br>• Modified Composite International Diagnostic Interview – Substance Abuse Module (CIDI-2 SAM)[54,55]
89-94	Sexual risk associated with substance use	Novel questions adapted from YRBS[42]

Assessment Battery

Demographic Information: Demographic information will include the participant's gender, race, ethnicity current grade in school, number of parents living in the home, highest level of parents' education. We are using parents' education level as a proxy measure for socio-economic status level.

Eligibility: Eligibility questions will be asked during the consent process before participants are consented into the study. This includes participant's age, ability to read English at a middle school level or higher, if a participant is currently pregnant, and verification that they have had a diagnosis of type 1 diabetes or a rheumatologic condition for at least one year.

<u>Health Information and Other Predictors of Substance Use</u>

Health information: Past medical history may inform a participant's decisions to use or abstain from alcohol and other drug use.[56,57] Youth may use illicit substances to self-medicate, or may avoid substances because of specific health concerns. We will ask participants to report past medical history, and to rate their overall health. We will use previously validated questions to assess these domains. A question on past medical history about being treated with prescribed medications for anxiety, depression, or ADHD was created novel for this battery.

Other factors that may affect substance use: Multiple components of the day to day life of youth may predict a relationship to substance use patterns. Predictors of interest for this project include social support, parental monitoring and communication, sleep, and family medical history.

Current mental health status: The PHQ-2 is a self-administered, 2-item scale for detecting depression using DSM-IV diagnostic criteria[45]. The PHQ-2 has been studied extensively in large populations and comes with considerable evidence for its validity. The PHQ-2 has been used extensively in primary care settings and adolescent populations. We will be administering the full PHQ-2 at baseline, 6 month follow up, and 12 month follow up.

The GAD-2 is a self-administered, 2 item scale to screen for anxiety disorders. It has high sensitivity and specificity for detecting generalized anxiety disorder and has been used in primary care settings[58]. We will be administering the full GAD-2 at baseline, 6 month follow up, and 12 month follow up.

<u>Alcohol, tobacco, marijuana, and use of other substances:</u> We will administer a series of questions about frequency of substance use and validate them against the criterion standard TLFB.

Substance use screen: We will administer the Screening to Brief Intervention (S2BI)[48] to ask about alcohol, tobacco, marijuana, prescription drug, and other substance use over the past 12 months. S2BI was developed at BCH with support from NIDA. In the original S2BI, questions on prescription drug use are asked only if patients answer yes to using alcohol, marijuana, or tobacco. In this study we will ask all participants about prescription drug use regardless of their use of other substances. Following the format of the S2BI, questions about other psychoactive substances including illegal drugs (such as cocaine or Ecstasy), inhalants (such as nitrous oxide), and herbs and synthetic drugs (such as salvia, "K2" or bath salts) will be asked only if participants answer yes to either alcohol, marijuana, or tobacco use.

Criterion standard for substance use disorder diagnosis: If participants answer no to past 12 month alcohol, marijuana, or tobacco use they will be asked a question about lifetime use of these substances. Questions about lifetime use were taken from the Computerized Diagnostic Interview Schedule for Children – 4 (DISC-IV).[59]

Alcohol safety screen: We will include questions about frequency and severity of alcohol use that will be used as safety flags. If a patient reports consuming 10 or more drinks containing alcohol in the past three months, or using sedatives in combination with alcohol, the RA will notify the primary clinician (See Adverse Event Criteria and Reporting Procedures)

Prescription medication misuse: Abuse of prescription medications among youth has risen dramatically in recent years. We will ask questions to assess if participants take medication as prescribed by their doctor. We will also assess medication diversion, with a specific interest in patients who are being prescribed stimulant medications. These questions were adapted from previous studies on prescription medication misuse among adolescents.[47]

Criterion standard substance use frequency measure: Each participant who reports any alcohol use will complete a 90-day Timeline Follow Back (TLFB) calendar that records frequency and quantity of alcohol use and frequency of marijuana use. The 90-day TLFB has been demonstrated to be both valid [60] and reliable [61] in adolescents. Participants will complete the TLFB on the computerized tablet following completion of the assessment battery. The TLFB is self-administered; participants will view an electronic calendar of the past 90 days and will input if they used marijuana on a given day and/or number of drinks consumed on a given day. If participants have not used marijuana or consumed a drink in the past 90 days, they will click through the calendar leaving the days blank. On the last screen they will be prompted to confirm that they did not select any days. The tool will be programmed to calculate total days of use and number of drinks.

Intermediary measures: We hypothesize that the following questions may serve as intermediary measures to predict long term substance use outcomes.

Time spent with alternative activities: Studies have shown that involvement in school based extra-curricular activities is generally related to less substance use among adolescents except in the case of athletic participation which has shown a positive relationship to substance use. We are interested in the number of hours participants spend per week engaged in different activities.[62]

Academic and extracurricular performance: Engagement, performance, and interest in academics and extracurricular activities, as well as disciplinary consequences may be indicators of current or future substance use. We will ask participants about personal consequences related to substance use. We are interested in consequences related to school such as academic performance, their commitment to school and extra-curricular activities, and how often they receive detention.

Perception of overscheduling: We will ask questions based on whether or not adolescents view themselves as overscheduled taken from the Measure of Perceived Over-Scheduling (MOPS) scale. We selected three items from the MOPS scale one from each factor measurement: pressure from external forces to participate in activities, feeling overwhelmed by participation, and preference for one's activities.[51]

Acceptability and interest in app/tools to monitor health behaviors/ Internet and social media use: We will ask questions about participant's willingness to use cellphone apps and tools to monitor health behaviors. This may provide information that can be used to inform development of health behavior interventions. Other factors that may also be of interest in informing interventions are the presence of alcohol and other drugs on social media websites and participant's use of the internet to search for information about alcohol or other drugs. These questions were created novel for this study.

Possible reasons to abstain from alcohol and marijuana use: We are interested in learning more about different reasons participants may choose to abstain from alcohol and marijuana use. We will ask participants to rate the importance of different statements as reasons to abstain from alcohol and marijuana. We will also assess whether perceived parental attitudes about smoking marijuana has an impact on marijuana use.

Knowledge about the impact of marijuana and alcohol on health: Brief interventions to prevent or reduce adolescent substance use often focuses on increasing youth knowledge of the harms associated with use, with the expectation that increased knowledge will lead to decreased use over time. We will ask novel questions to assess participants' knowledge on the effects of substance use.

Perceived risk of harm from substance use: In nationally representative surveys, perceived risk of harm related to substance use is strongly associated with marijuana use on a population level, though less is known about the predictive validity on an individual level. We will ask participants questions regarding perceived risk of harm of alcohol, marijuana, tobacco and prescription medications in order to describe associations between perceived risk of harm and substance use trajectories.

Perceived availability of alcohol and marijuana: Increased availability of alcohol has been associated with increased alcohol use and related problems in youth.[63] To look at perceived availability of substances and the correlation with substance use on an individual level, we will ask a question about how difficult it would be for participants to get alcohol and marijuana if they wanted some. These questions were taken from the Monitoring the Future study.

Perceived likelihood of future substance use: We will use questions from Monitoring the Future that ask about likelihood of future use validated in a longitudinal sample to determine whether this question is a good proxy measure of future use. We will ask participant about their likelihood of substance use three months from when they are completing the assessment battery and analyze responses against reports of actual substance use collected longitudinally.

Receipt of healthcare advice about substances from a medical professional[11]: To determine adolescents' experience of physician screening we will whether a clinician has asked about substance use and willingness to talk to a clinician or learn more about how alcohol use can affect health. We will use questions that were piloted by the research team in a previous project (NIAAA number).

Confidence in refusal skills: We are interested in learning about participant's confidence in their ability to refuse substances from friends. An adolescent's peers can have an influence on their behavior or substance use.[64] We will include one novel question in our battery to assess participant's perceived self-efficacy.

Grit: Perseverance and passion for long term goals may predict substance use trajectories[36]. We will be administering the 8-Item Grit Scale, which is a two factor model that measures consistency of interest and perseverance of effort. The Grit Scale has been used in research settings and has been validated in both adult and adolescent populations[65]. In these populations it has been used to look at relationships in educational attainment, career changes, and predicted GPA.[65] We will include all 8 items from the Short Grit Scale in our assessment battery.

Future goals for after high school: Goals for the future may predict substance use by high school students. This has been studied in adjudicated adolescents, where positive future orientation was associated with lower levels of substance use.[66] There has been little research in goal setting and future orientation as a predictor for other populations of adolescents. We would like to include a section on future goals in our assessment battery to study it further as a predictor for substance use. We will ask participants whether they have plans for their future after high school.

<u>Substance specific patient centered outcomes.</u>

Problems and consequences associated with alcohol and marijuana use: Standard measures of consequences related to substance use typically mix all substances together, creating a composite scale. For this project, we will separately determine the most common consequences reported by

adolescents associated with alcohol use and marijuana use. Using the Personal Experience Screening Questionnaire (PESQ), we will ask participants substance specific outcome questions to determine whether alcohol and marijuana have different patterns of outcomes.

Sexual risk associated with substance use: Risky sexual contact is commonly associated with alcohol use.[67] Using questions adapted from the Youth Risk Behavior Survey[42], we will assess if a participant has ever engaged in sexual contact Specifically we are interested in whether there are different relationships and perceived concerns between alcohol and/or marijuana and unprotected sex.

Disease specific health questions

Disease management: We will ask a set of questions to learn more about current disease status and how patients manage their chronic medical conditions. Diabetes specific questions include use of an insulin pump, use of a glucose monitor, number of times he/she has had their hemoglobin A1c (HbA1c) checked by a doctor in the past 12 months, last HbA1c reading, and number of times they check their blood sugar daily. Rheumatologic condition specific questions include a pain scale and how often they have experienced morning stiffness in the past 30 days.

Disease severity: We will ask patients with type 1 diabetes about episodes of severe hyperglycemia and hypoglycemia and patients with rheumatological conditions about frequency of disease flares to learn more about their medical condition.

Intervention:

Those in the intervention arm will receive a computerized brief intervention composed of tailored feedback and psycho-education. Content in the intervention will focus on health promotion and will deliver positive messages about health.

The Co-PIs previously conducted a qualitative study (Validating NIAAA's Brief Screening Guide in Youth with Chronic Medical Conditions) among YCMC receiving care at BCH. The goal of the study was to understand how they navigate their chronic medical condition in general and in social situations, situations in which they have used or not used substances, and the quality of messages they have received about their health. The core domains this intervention will focus on were adapted from data collected during the qualitative study and a literature review on the topic. The domains were refined and then pilot tested with a sample of patients who participated in the aforementioned qualitative study.

Domains included in the intervention are 1) disclosure of disease to peers and resistance skills; 2) adolescent development; 3) medication adherence; 4) and disease specific knowledge. The topic area will include information on science or key findings in research, narratives from peers on their experiences, and actionable items or key messages that reinforce the topic. Quotes from youth with chronic medical conditions collected from the previous qualitative study were included as part of the narrative sections to provide participants with messages from peers.

Disclosure of disease to peers and resistance skills: Studies have shown that peer support from close friends had an impact on stressors such as adjusting to a chronic condition or coping with a difficult medical treatment in youth with chronic medical conditions.[68] We are also interested in including a section on refusal skills in out intervention. Findings suggest that resistance skills training may be especially effective before the onset of behavior as a primary prevention tool.[69] Youth who lack refusal skills are more likely to engage in alcohol use in early adolescence and this trend persists into later adolescence as well.[70] These two themes will be combined into a section about encouraging communication with peers if they have not already done so and increasing comfort in doing so. It also

includes recommendations to use close friends in building resistance skills, having “sober buddies” and in supporting decisions not to use substances.

Adolescent Development: Adolescents growing up with a chronic medical condition face unique challenges and burdens compared to other adolescents. Findings from our qualitative study showed that many adolescents felt their condition and management of their health made them feel more responsible and mature compared to others their age. This section will focus on acknowledging that while YCMC may feel burdened by their condition there may be positive ways in which their condition has changed their life, including increased maturity and resilience.

Medication Adherence: A previous study by the Co-PIs found that high school youth who reported past year alcohol use were 1.79 times as likely to report they forgot to take their medications and 1.61 times as likely to report skipping their medications “always/ often/sometimes” over the past 30 days than youth who did not report past year alcohol use.[71] This section will provide information around medication adherence and the possible effects of substance use on adherence.

Disease Knowledge: A previous study by the Co-PIs also showed that knowledge about whether alcohol can interfere with medications or laboratory tests used to treat chronic conditions was low among YCMC. Alcohol use among youth with type 1 diabetes can pose serious health risks such as hypoglycemia.[72] A common theme in the qualitative work by the Co-PIs was a preference from adolescents for direct and hones information about the disease-specific risks of substance use without “any gray areas” or “sugarcoating” information[73]. The knowledge section will focus on presenting information about how alcohol is processed in the body and how this may impact chronic conditions or the effectiveness of the medications used to treat chronic conditions.

Control: Those in the control arm will receive information/brief advice around internet safety.

Information for the internet safety was adapted from existing curriculum available through Netsmartz and KidsHealth.org. The core topic around internet safety will focus on how youth can protect their online presence and increase awareness of their “digital footprint”. We will provide information on the importance of being safe about posting online, possible consequences of what posting personal information, and protecting online privacy.

Follow-up Assessment: We will follow up with the recruited sample 6 months and 12 months post baseline. We will administer an abbreviated Assessment Battery, either online or via phone, with a focus on changes in behavior since baseline to measure substance use and outcome trajectories over time. The assessment batteries administered at 6 and twelve months post baseline are attached to this protocol.

Parent Assessment Battery

We will recruit parents of participants enrolled in the trial to complete an assessment battery at baseline. We will ask parents questions to ascertain their opinions around a variety of health related issues and how they relate to their child with a chronic condition. Topics will include disease burden, mental health, care transition, knowledge and attitudes around substance use, social media use, and use of marijuana as medicine. Due to the a lack of studies that have looked specifically at parents of youth with chronic medical conditions and many of these topics, a number of our questions are novel, however questions in this survey have been pilot tested a well received in a related anonymous survey of parents whose children have chronic medical conditions (IRB-P00017232).

**Q #s**	**Measure/ Domain**	**Name of tool**
**1. Eligibility and Child Demographics**		

1	Eligibility - Child between 14-17 with type 1 diabetes	Novel
2, 4	Total number of children and adults in household	Novel
3	Total number of children in household with chronic medical condition	Novel
**2. Parent management of child's diabetes/rheumatic condition and other behaviors**		
5	Managing child's diabetes/rheumatic condition	Problem Areas in Diabetes PAID – Parent Proxy revised for rheumatic conditions survey[74]
6-7	Transitioning of care	Novel
8	Provider speaks alone	National Survey of Children with Special Healthcare Needs[40]
9	Patient Provider Communication	Novel
10-11	Using the internet for online support groups	Novel
12	Child's social media use and parental monitoring	CASA Columbia[52]
**3. Intermediary Measures**		
13, 15 (9 items each) 14,16	Perceived Risk Alcohol/Marijuana	Novel
17 (4 items)	Perceptions on Marijuana as Medicine	Novel
18 (8 items)	Child's use	Novel
19, 22 (12 items)	Concern about alcohol/marijuana on CMC	Novel
20-21, 23-24	Physical health problems or complications due to substance use	
26-28	Discussed risks of substance use with child	Northeast Center for Healthy Communities (NCHC) modified
26	Enough info. To talk to child about substance use /CMC?	Novel
30-32	Medical provider discussed SU with child	Novel
33-34	Best way for child to learn about SU and CMC	Novel
**4. Parent Information**		
45-47	Own use (Alcohol, marijuana, tobacco)	Novel
36	Own state legislature on MJ	Novel
35, 37-44	Demographic Info (Type of guardian i.e. parent, grandparent, gender, ethnicity, race, level of education, marital status, household income)	Novel

Study Timeline (as applicable)

Activity	Year 1				Year 2				Year 3				Year 4			
Develop measures/ Develop intervention	X	X	X													
Pre-Test measurement battery			X	X												
Develop/User test electronic screening			X	X												

algorithm																
Recruit/consent participants					X	X	X	X	X	X						
Follow up at 6 and 12 months						X	X	X	X	X	X	X	X	X		
Data Analysis															X	X
Report results – creation of Resource Guide																X

E. Adverse Event Criteria and Reporting Procedures

We do not anticipate any adverse events as a result of this study. The greatest risk is potential breach of confidentiality regarding sensitive information (e.g., use of drugs and alcohol). To protect against this possibility, we will use randomly generated study identifiers, created by a computer, in all surveys, transcripts and databases. Code keys that link the study identifier to the subject identity (medical record number, name, and telephone number) will be maintained throughout the study period. The keys will be stored and locked (password-protected) in a secure location.

Although not induced by study participation, serious substance use may be detected during completion of the assessment batteries. We will follow the same guidelines for assessment of risk related to substance use that has been used in protocols previously approved by the BCH institutional review board. If a participant responds “yes” to consuming 10 or more alcoholic drinks in one occasion within the past 3 months, using sedatives (such as benzodiazepine, opioids, barbiturates, or pain medication) in combination with alcohol, using stimulants (such as Adderall or Ritalin) in combination with alcohol, or using illegal drugs other than marijuana (such as cocaine, or Ecstasy) monthly, the RA will notify the primary clinician. At baseline, the RA will locate the primary clinician in the clinic following completion of the assessment battery. The primary clinician will be able to re-interview the patient, as clinically warranted, and determine what further action should be taken. The RA will also notify the primary clinician if there is any indication or concerns about the participant’s risk for imminent harm. We will ask the primary clinician to sign our Alcohol Safety Sheet (see attached) to indicate that he/she has been notified. We will tell participants that we cannot make absolute assurances or provide them with specific guidelines on parental notification. We will explain that we will only inform their clinician and that the clinician will decide if a participant’s parents should be notified based on the best clinical judgment at the time. We will assure participants that we will make every effort *to inform them before the fact* when breach of confidentiality is required, to include them in a discussion regarding the exact details that are to be released and to release as few details as possible to ensure the safety of the adolescent (or other person).

Although not induced by study participation, depressive symptoms may also be detected during the baseline study assessment through completion of the PHQ-2. If a participant scores 3 or greater on the PHQ-2, indicating a non-acute need for further assessment, the RA will inform the participant that we will be notifying his/her provider, who may follow up with them by phone, or at their next appointment. We will notify the participant’s provider of the PHQ-2 score via email by the end of the day. We will ask that the provider reply to our e-mail to confirm that he or she has been notified of the safety concern. The provider will then address the mental health safety concern according to standard clinical practice. If the RA does not receive confirmation of receipt from the clinician, the RA will follow up with a phone call and/or page to ensure that the message is received. Both Principal Investigators (Levy and Weitzman) will be alerted of all safety concerns via email.

At 6 and 12 months follow up safety concerns may also arise through completion of the assessment batteries. We will address follow up safety flags through e-mails to the participant’s

specialty care clinician. We will notify the clinician via e-mail if a participant has a PHQ-2 score of 3 or higher (indicating a non-acute need for further assessment), or if serious substance use is detected (high volume alcohol consumption or use of sedatives in combination), and request confirmation that the participant's clinician has received the notification and is using standard clinical protocol to determine appropriate follow-up and/or referrals. If we do not receive confirmation of receipt from the clinician, we will follow up with a phone call and/or page to ensure that the message is received. We will provide email notification to clinicians of any safety flags raised within 48 hours of survey completion.

We will provide participants with contact information for their primary and specialty care providers if they wish to talk to a health professional or obtain help regarding substance use and/or mental health.

A text message or email will also be sent to participants via BCH's outlook email system with the names and telephone numbers of two resources outside of BCH they may call if they wish to talk to a professional or gain help regarding substance use and/or mental health. If the participant does not readily know their cell phone carrier, which is required for sending the text message via email, an external website will be utilized to identify the cell phone carrier. Participant will also be provided verbally with the contact numbers for these resources upon request, or if they do not wish to receive a text message, but would still like the information.

It is also possible, but unlikely, that some participants could become upset by items in the measurement battery or information presented in the intervention or control arms. We include statements at the start that participants may skip questions that they are uncomfortable answering, that they may stop the study at any time, and that they may speak with the RA after completing the measurement if they have any questions or concerns about the questions asked or the material presented.

Given the nature of the study, we anticipate few if any adverse events. If we determine during the course of the study that an adolescent or someone else is at serious risk of harm, we will notify their provider. We will inform adolescents of this before we ask them to assent to participation in the study. We do not anticipate any serious adverse events, such as medical complications or deaths, as a result of this project.

F. Data Management Methods

Drs. Levy and Weitzman will be responsible for data and safety monitoring (DSM). We will meet frequently (at least weekly) with the study RA's to monitor recruitment and work intensely with the clinic if we are falling behind study targets. We will periodically analyze the demographic characteristics of the study sample to ensure that both the control group and intervention group are similar. We will also analyze the demographic characteristics of the study sample to ensure that it reflects the clinic populations at large.

All study-related recruitment data and assessments will be entered into the data management system which has been used for other projects conducted by the PIs. This system has been designed with the following features: (1) A menu-driven study management system that tracks each study participant's status; (2) Data integrity checks using form-oriented data entry screens; 3) User prompts for missing, out-of-range and illogical responses; (4) Regular reports of subject accrual and pre-specified aggregate data; (5) Secure master files; (6) Daily back-up, data security and confidentiality with access to the system and permitted functions (e.g., making changes to data) controlled by password.

G. Quality Control Method

The research team will meet weekly in the preparatory and implementation phases before recruitment starts. Collaborators and consultants will be invited to join team meetings quarterly or more if necessary. Drs. Levy and Weitzman will review study progress at least monthly with the RAs. All data will be saved on a secure, existing BCH server set up for this purpose. We will review recruitment and retention rates, ensure data integrity, troubleshoot problems, and address any deviations from the protocol that arise. Minutes will be recorded and circulated to the team following every meeting. We will perform a data integrity check after approximately 10% of the sample has been recruited in order to verify that all systems are working properly.

H. Data Analysis Plan

All analyses will be conducted under the principle of intention to treat; all participants will be analyzed as members of the groups to which they were originally randomized to. To assess baseline equivalence between intervention group and control group, we will compare distribution of demographic characteristics using $\chi 2$ test or Fisher exact test for categorical variables and t-test or Wilcoxon sign rank test for continuous variables. We will also evaluate equivalence among those lost to follow-up versus those retained by comparing demographics and outcomes among those lost and those with complete follow-up.

Our primary outcome variables will be initiation and escalation of substance use. Initiation of use will be defined as any new use of substances at follow-up among those reporting no past-12-month use at baseline. For alcohol use, escalation will be defined as, among drinkers, any increase in number of drinks (measured by TLFB) from baseline to follow-up. For marijuana use, escalation will be defined as, among users, any increase in days of smoking marijuana (measured by TLFB) from baseline to follow-up.

We will assess the magnitude and direction of the effects of hypothesized predictors on substance use. Specifically, we will examine the association between each factor and past year substance use, past 90 days use and number of drinks measured by TLFB. Multivariate logistic/linear regression, adjusting for demographic characteristics, will be performed.

To evaluate the effect of intervention on substance use at 6- and 12-month follow-up, we will perform multivariate logistic regression adjusting for demographics, predictors of substance use and baseline knowledge. Separate models will be used to evaluate effects at 6- and 12-month. Adjusted odds ratios will be computed for comparison between intervention and control groups.

We will also evaluate how well the change of intermediary measures predicts the change of substances use. We will use multiple logistic regression incorporating all intermediary variables. Separate models will be used to assess the associations at 6- and 12-month.

I. Statistical Power and Sample Considerations

We will recruit a convenience sample of 450 adolescent patients (n=450) and expect 300 of them will complete the entire study. With total sample size of 300 youth, there is adequate power (.80) to detect significant effects ($p<.05$) for adjusted odds ratios of ≥ 2.0, defined as the likelihood that control versus intervention participants initiate or escalate their substance use behaviors from baseline to follow-up, across prevalence levels in the intervention group that range from .20 to .55.

J. Study Organization Sharon Levy, MD, MPH and Elissa Weitzman, ScD, MSc will direct all aspects of the project. They will conduct interviews with clinic providers and patients to determine additional study measurements and provider advice pertinent to their patients' chronic conditions. They will plan, direct and monitor study recruitment. They will be responsible for the training and work of the research

coordinators and research assistant. They will ensure approval of the protocol through the Children's Hospital Committee on Clinical Investigations (IRB), oversee the project funds, and ensure completion of required reports to the Hilton Foundation and the IRB. They will oversee data analysis and preparation of study manuscripts and dissemination of study findings.

- Kate Garvey, MD is a pediatric diabetologist at the Endocrinology clinic at BCH. She will serve as clinical collaborator to provide feedback on the assessment battery measures. Dr. Garvey will also serve as the point of contact for the Endocrinology clinic. She will ensure integration of this study into clinical workflow of the Endocrinology clinic, and will provide guidance on recruitment, analysis, and reporting of validation data.
- Fatma Dedeoglu, MD is a pediatric rheumatologist in the Rheumatology clinic at BCH. She will serve as a clinical collaborator to provide feedback on the assessment battery measures. Dr. Dedeoglu will also serve as the point of contact for the Rheumatology clinic. She will ensure integration of this study into clinical workflow of the Rheumatology clinic, and will provide guidance on recruitment, analysis, and reporting of validation data.
- Lauren Wisk, PhD will provide national and contextualizing information about ATOD risk for feedback of screening results to intervention study participants and providers. She will additionally assist with data analysis.
- Erin Qian Huang, MPH will assist with finalizing the assessment measures and oversee converting them to electronic data capture format. They will monitor study recruitment, and assist with quantitative data analysis and preparation of study manuscripts.
- Julie Lunstead, MPH will oversee the hiring and training of the research coordinators and research assistants and preparation of IRB application and yearly renewal. She will manage the project funds and assist with preparation of study reports, data analysis and reporting of results.

Lydia Shrier, MD, MPH; and Elizabeth Harstad, MD will serve as clinic collaborators to provide feedback on the assessment battery measures.

References

1. Van Cleave J, Gortmaker SL, Perrin JM. Dynamics of obesity and chronic health conditions among children and youth. *JAMA*. 2010;303(7):623-630. doi:303/7/623 [pii] 10.1001/jama.2010.104.

2. Halfon N, Newacheck PW. Evolving notions of childhood chronic illness. *JAMA*. 2010;303(7):665-666. http://www.ncbi.nlm.nih.gov/pubmed/20159877. Accessed October 7, 2014.

3. Institute of Medicine. *Living Well with Chronic Illness: A Call for Public Health Action*. Washington, D.C.: National Academies Press; 2012. http://www.iom.edu/Reports/2012/Living-Well-with-Chronic-Illness.aspx.

4. Bender BG, Rand C. Medication non-adherence and asthma treatment cost. *Curr Opin Allergy Clin Immunol*. 2004;4(3):191-195. doi:00130832-200406000-00009 [pii].

5. Kann L, Kinchen S, Shanklin S, et al. *Morbidity and Mortality Weekly Report: Youth Risk Behavior Surveillance—United States 2013, Surveillance Summary No. 63 (No.4); 1-172*. Vol 63.; 2014. http://www.cdc.gov/mmwr/pdf/ss/ss6304.pdf?utm_source=rss&utm_medium=rss&utm_campaign=youth-risk-behavior-surveillance-united-states-2013-pdf.

6. Hingson RW, Heeren T, Winter MR. Age at drinking onset and alcohol dependence: age at onset, duration, and severity. *Arch Pediatr Adolesc Med*. 2006;160(7):739-746. http://www.ncbi.nlm.nih.gov/entrez/query.fcgi?cmd=Retrieve&db=PubMed&dopt=Citation&list_uids=16818840.

7. Center for Disease Control and Prevention. Fact Sheets: Underage Drinking - Alcohol. http://www.cdc.gov/alcohol/fact-sheets/underage-drinking.htm. Accessed August 20, 2014.

8. Substance Abuse and Mental Health Services Administration (SAMHSA). *Results from the 2009 National Survey on Drug Use and Health: Volume I. Summary of National Findings*. Vol 1. Summary. Rockville, MD ; 2010.

9. Hingson RW, Zha W. Age of drinking onset, alcohol use disorders, frequent heavy drinking, and unintentionally injuring oneself and others after drinking. *Pediatrics*. 2009;123(6):1477-1484. doi:123/6/1477 [pii]10.1542/peds.2008-2176.

10. Volkow ND, Baler RD, Compton WM, Weiss SRB. Adverse Health Effects of Marijuana Use. *N Engl J Med*. 2014;370(23):2219-2227. http://www.nejm.org/doi/full/10.1056/NEJMra1402309?query=featured_home&. Accessed July 30, 2014.

11. Weitzman ER, Nelson TF. College student binge drinking and the “prevention paradox”: implications for prevention and harm reduction. *J Drug Educ*. 2004;34(3):247-265. http://www.ncbi.nlm.nih.gov/pubmed/15648886.

12. Barry KL, Blow FC, Willenbring ML, McCormick R, Brockmann LM, Visnic S. Use of Alcohol Screening and Brief Interventions in Primary Care Settings: Implementation and Barriers. *Subst Abus*. 2004;25(1):27-36. doi:10.1300/J465v25n01_05.

13. Substance Abuse and Mental Health Services Administration. About Screening, Brief Intervention, and Referral to Treatment (SBIRT). http://www.samhsa.gov/sbirt/about. Published 2015.

14. Levy S, Williams JF, Knight JR. Screening, brief intervention, and referral to treatment for adolescents: Companion clinical case. *J Addict Med*. 2008;2(4):222-226. http://familymed.uthscsa.edu/sstart/documents/SBIRT Adolescent Case.pdf.

15. Levy SJL, Kokotailo PK. Substance use screening, brief intervention, and referral to treatment for pediatricians. *Pediatrics*. 2011;128(5):e1330-e1340. doi:10.1542/peds.2011-1754.

16. Quanbeck A, Lang K, Enami K, Brown RL. A cost-benefit analysis of Wisconsin’s screening, brief intervention, and referral to treatment program: adding the employer's perspective. *WMJ*. 2010;109(1):9-14.

17. Fleming MF, Mundt MP, French MT, Manwell LB, Stauffacher EA, Barry KL. Brief physician advice for problem drinkers: long-term efficacy and benefit-cost analysis. *Alcohol Clin Exp Res*. 2002;26(1):36-43.

18. National Institute on Alcohol Abuse and Alcoholism. *Alcohol Screening and Brief Intervention for Youth: A Practitioner's Guide.* NIH Publication No. 11-7805; 2011. http://pubs.niaaa.nih.gov/publications/Practitioner/YouthGuide/YouthGuide.pdf.

19. Substance Abuse and Mental Health Services Administration (SAMHSA). Motivational Interviewing. Training. http://www.samhsa.gov/co-occurring/topics/training/motivational.aspx.

20. Massachusetts Department of Public Health Bureau of Substance Abuse Services. Provider Guide: Adolescent Screening, Brief Intervention, and Referral to Treatment - Using the CRAFFT Screening Tool. 2009.

21. Elster AB, Kuznets NJ. AMA Guidelines for Adolescent Preventive Services (GAPS). 1994.

22. Hagan JF, Shaw JS, Duncan P. Bright Futures Guidelines for Health Supervision of Infants, Children, and Adolescents: Third Edition. 2008.

23. U.S. Preventive Services Task Force. *Screening and Behavioral Counseling Interventions in Primary Care to Reduce Alcohol Misuse: Recommendation Statement. AHRQ Publication No. 12-05171-EF-3*.; 2013. http://www.uspreventiveservicestaskforce.org/uspstf12/alcmisuse/alcmisuserfinalrs.htm.

24. Harris SK, Csemy L, Sherritt L, et al. Computer-facilitated substance use screening and brief advice for teens in primary care: an international trial. *Pediatrics*. 2012;129(6):1072-1082. doi:peds.2011-1624 [pii]10.1542/peds.2011-1624.

25. Harris SK, Herr-Zaya K, Weinstein Z, et al. Results of a statewide survey of adolescent substance use screening rates and practices in primary care. *Subst Abus*. 2012;33(4):321-326. doi:10.1080/08897077.2011.645950.

26. Levy SJL, Kokotailo PK. Substance use screening, brief intervention, and referral to treatment for pediatricians. *Pediatrics*. 2011;128(5):e1330-e1340. doi:10.1542/peds.2011-1754.

27. Schram P, Harris SK, Van Hook S, et al. Implementing Adolescent SBIRT Education in a Pediatric Residency Curriculum. *Subst Abus*. July 2014. http://www.ncbi.nlm.nih.gov/pubmed/25036267. Accessed October 16, 2014.

28. Weitzman ER, Kaci L, Quinn M, Mandl KD. Helping high-risk youth move through high-risk periods: personally controlled health records for improving social and health care transitions. *J diabetes Sci Technol*. 2011;5(1):47-54.

29. Knight JR, Shrier LA, Bravender TD, Farrell M, Vander Bilt J, Shaffer HJ. A new brief screen for adolescent substance abuse. *Arch Pediatr Adolesc Med*. 1999;153(6):591-596.

30. Shaffer D, Fisher P, Lucas CP, Dulcan MK, Schwab-Stone ME. NIMH Diagnostic Interview Schedule for Children Version IV (NIMH DISC-IV): description, differences from previous versions, and reliability of some common diagnoses. *J Am Acad Child Adolesc Psychiatry*. 2000;39(1):28-38.

31. MacKey K, Parchman ML, Leykum LK, Lanham HJ, Noël PH, Zeber JE. Impact of the Chronic Care Model on medication adherence when patients perceive cost as a barrier. *Prim Care Diabetes*. 2012;6(2):137-142. doi:10.1016/j.pcd.2011.12.004.

32. Weitzman ER, Kelemen S, Kaci L, Mandl KD. Willingness to share personal health record data for care improvement and public health: a survey of experienced personal health record users. *BMC Med Inform Decis Mak*.

33. Weitzman ER, Adida B, Kelemen S, Mandl KD. Sharing data for public health research by members of an international online diabetes social network. *PLoS One*. 2011;6(4).

34. Weitzman ER, Kelemen S, Mandl KD. Surveillance of an Online Social Network to Assess Population-level Diabetes Health Status and Healthcare Quality. *Online J Public Health Inform*. 2011;3(3). http://www.pubmedcentral.nih.gov/articlerender.fcgi?artid=3615790&tool=pmcentrez&rendertype=abstract.

35. McCabe SE, Diez A, Boyd CJ, Nelson TF, Weitzman ER. Comparing web and mail responses in a mixed mode survey in college alcohol use research. *Addict Behav*. 2006;31(9):1619-1627. doi:10.1016/j.addbeh.2005.12.009.

36. Duckworth AL, Peterson C, Matthews MD, Kelly DR. Grit: perseverance and passion for long-term goals. *J Pers Soc Psychol*. 2007;92(6):1087-1101. doi:10.1037/0022-3514.92.6.1087.

37. Kroenke K, Spitzer RL, Williams JBW. The PHQ-9. *J Gen Intern Med*. 2001;16(9):606-613. doi:10.1046/j.1525-1497.2001.016009606.x.

38. Winters KC. Development of an adolescent alcohol and other drug abuse screening scale: Personal experience screening questionnaire. *Addict Behav*. 1992;17:479-490.

39. National Institute of Health. PROMIS Overview. http://www.nihpromis.org/about/overview?AspxAutoDetectCookieSupport=1. Published 2015.

40. Child and Adolescent Health Measurement Initiative. *National Survey of Children's Health (NSCH) 2011/12 : Guide to Topics & Questions Asked*.; 2012. http://childhealthdata.org/docs/drc/2011-12-guide-to-topics-questions-draft.pdf?sfvrsn=4.

41. Weissberg-Benchell J, Antisdel-Lomaglio J. Diabetes-specific emotional distress among adolescents: feasibility, reliability, and validity of the problem areas in diabetes-teen version. *Pediatr Diabetes*. 2011;12(4pt1):341-344. doi:10.1111/j.1399-5448.2010.00720.x.

42. Center for Disease Control and Prevention. *2015 State and Local Youth Risk Behavior Survey*.; 2015. http://www.cdc.gov/healthyyouth/yrbs/pdf/questionnaire/2015_hs_questionnaire.pdf.

43. Tobler AL, Komro KA. Trajectories or parental monitoring and communication and effects on drug use among urban young adolescents. *J Adolesc Health*. 2010;46(6):560-568. doi:10.1016/j.jadohealth.2009.12.008.

44. Center for HIV Identification Prevention and Treatment Services. *Social Support for Adolescents Scale (SSAS)*.; 2012. http://chipts.ucla.edu/wp-content/uploads/downloads/2012/01/Social-Support-for-Adolescents-Scale-_SSAS_.pdf.

45. Kroenke K, Spitzer RL, Williams JBW. The Patient Health Questionnaire-2: validity of a two-item depression screener. *Med Care*. 2003;41(11):1284-1292. doi:10.1097/01.MLR.0000093487.78664.3C.

46. Kroenke K, Spitzer RL, Williams JBW, Monahan PO, Löwe B. Anxiety disorders in primary care: prevalence, impairment, comorbidity, and detection. *Ann Intern Med*. 2007;146(5):317-325. http://www.ncbi.nlm.nih.gov/pubmed/17339617. Accessed September 22, 2015.

47. McCabe SE, Teter CJ, Boyd CJ. Medical use, illicit use and diversion of prescription stimulant medication. *J Psychoactive Drugs*. 2006;38(1):43-56. doi:10.1080/02791072.2006.10399827.

48. Levy S, Weiss R, Sherritt L, et al. An Electronic Screen for Triaging Adolescent Substance Use by Risk Levels. *JAMA Pediatr*. 2014;168(9):822-828. doi:10.1001/jamapediatrics.2014.774.

49. Johnston LD, O'Malley PM, Miech RA, Bachman JG, Schulenberg JE. *Monitoring the Future National Results on Drug Use: 1975-2013: Overview, Key Findings on Adolescent Drug Use*. Ann Arbor; 2014.

50. Sobell LC, Sobell MB. Timeline follow-back: A technique for assessing self-reported alcohol consumption. In: Litten R, Allen J, eds. *Measuring Alcohol Consumption: Psychological and Biolgical Methods*. ; 1992:41-72.

51. Pollock A. The Development of the Measure of Perceived Overscheduling (MOPS). *PCOM Psychol Diss*. 2010:Paper 172. http://digitalcommons.pcom.edu/psychology_dissertations/172. Accessed July 21, 2015.

52. The National Center on Addiction and Substance Abuse. *National Survey of American Attitudes on Substance Abuse XVII : Teens*. New York, NY; 2012.

53. Cottler LB, Robins LN, Helzer JE. The reliability of the CIDI-SAM: a comprehensive substance abuse interview. *Br J Addict*. 1989;84(7):801-814.

54. Kelly SM, Gryczynski J, Mitchell SG, Kirk A, O’Grady KE, Schwartz RP. Validity of brief screening instrument for adolescent tobacco, alcohol, and drug use. *Pediatrics*. 2014;133(5):819-826. http://pediatrics.aappublications.org/content/133/5/819.short. Accessed November 24, 2014.

55. Kelly SM, Gryczynski J, Mitchell SG, Kirk A, O’Grady KE, Schwartz RP. Concordance between DSM-5 and DSM-IV nicotine, alcohol, and cannabis use disorder diagnoses among pediatric patients. *Drug Alcohol Depend*. 2014;140:213-216. http://www.sciencedirect.com/science/article/pii/S0376871614008266. Accessed November 11, 2014.

56. Surís J-C, Michaud P-A, Akre C, Sawyer SM. Health risk behaviors in adolescents with chronic conditions. *Pediatrics*. 2008;122(5):e1113-e1118. doi:10.1542/peds.2008-1479.

57. Increased Prevalence of Risk Behaviors Reported by Chronically Ill Adolescents. *NEJM J Watch*. 2008;2008. doi:10.1056/PA200812100000003.

58. Skapinakis P. The 2-item Generalized Anxiety Disorder scale had high sensitivity and specificity for detecting GAD in primary care. *Evid Based Med*. 2007;12(5):149. doi:10.1136/ebm.12.5.149.

59. Schwab-Stone ME, Shaffer D, Dulcan MK, et al. Criterion validity of the NIMH Diagnostic Interview Schedule for Children Version 2.3 (DISC-2.3). *J Am Acad Child Adolesc Psychiatry*. 1996;35(7):878-888. doi:10.1097/00004583-199607000-00013.

60. Knight JR, Sherritt L, Shrier LA, Harris SK, Chang G. Validity of the CRAFFT substance abuse screening test among adolescent clinic patients. *Arch Pediatr Adolesc Med*. 2002;156(6):607-614.

61. Levy S, Sherritt L, Harris SK, et al. Test-retest reliability of adolescents’ self-report of substance use. *Alcohol Clin Exp Res*. 2004;28(8):1236-1241.

62. Farb AF, Matjasko JL. Recent advances in research on school-based extracurricular activities and adolescent development. *Dev Rev*. 2012;32(1):1-48. doi:10.1016/j.dr.2011.10.001.

63. Treno AJ, Ponicki WR, Remer LG, Gruenewald PJ. Alcohol outlets, youth drinking, and self-reported ease of access to alcohol: a constraints and opportunities approach. *Alcohol Clin Exp Res*. 2008;32(8):1372-1379. doi:10.1111/j.1530-0277.2008.00708.x.

64. Allen JP, Chango J, Szwedo D, Schad M, Marston E. Predictors of susceptibility to peer influence regarding substance use in adolescence. *Child Dev*. 83(1):337-350. doi:10.1111/j.1467-8624.2011.01682.x.

65. Duckworth AL, Quinn PD. Development and validation of the short grit scale (grit-s). *J Pers Assess*. 2009;91(2):166-174. doi:10.1080/00223890802634290.

66. Robbins RN, Bryan A. Relationships Between Future Orientation, Impulsive Sensation Seeking, and Risk Behavior Among Adjudicated Adolescents. *J Adolesc Res*. 2004;19(4):428-445. doi:10.1177/0743558403258860.

67. Cooper ML. Alcohol use and risky sexual behavior among college students and youth: evaluating the evidence. *J Stud Alcohol Suppl*. 2002;(14):101-117. http://www.ncbi.nlm.nih.gov/pubmed/12022716. Accessed August 5, 2015.

68. La Greca AM, Bearman KJ, Moore H. Peer relations of youth with pediatric conditions and health risks: promoting social support and healthy lifestyles. *J Dev Behav Pediatr*. 2002;23(4):271-280. http://www.ncbi.nlm.nih.gov/pubmed/12177575. Accessed January 6, 2016.

69. Komro KA, Perry CL, Williams CL, Stigler MH, Farbakhsh K, Veblen-Mortenson S. How did Project Northland reduce alcohol use among young adolescents? Analysis of mediating variables. *Health Educ Res*. 2001;16(1):59-70. http://www.ncbi.nlm.nih.gov/pubmed/11252284. Accessed January 7, 2016.

70. Scheier LM, Botvin GJ, Diaz T, Griffin KW. Social skills, competence, and drug refusal efficacy as predictors of adolescent alcohol use. *J Drug Educ*. 1999;29(3):251-278. http://www.ncbi.nlm.nih.gov/pubmed/10645126. Accessed January 7, 2016.

71. Weitzman ER, Ziemnik RE, Huang Q, Levy S. Alcohol and Marijuana Use and Risks for Treatment Non-Adherence Among Medically Vulnerable Youth. *Pediatrics*. (in press).

72. Canadian Diabetes Association. *Alcohol + Diabetes Frequency Asked Questions for Healthcare Professionals Nutrition Guidelines Implementation Subcommittee Question*.; 2006. http://www.mountsinai.on.ca/care/lscd/sweet-talk-1/images-and-resources/AlcoholandDiabetes.pdf.

73. Weitzman ER, Salimian PK, Rabinow L, Levy S. Shaped By My Disease: Perspectives on Substance Use Shared by Youth with Chronic Medical Conditions. In: *National Pediatric Academic Societies Meeting*. ; 2015.

74. Markowitz JT, Volkening LK, Butler DA, Antisdel-Lomaglio J, Anderson BJ, Laffel LMB. Re-examining a measure of diabetes-related burden in parents of young people with Type 1 diabetes: the Problem Areas in Diabetes Survey - Parent Revised version (PAID-PR). *Diabet Med*. 2012;29(4):526-530. doi:10.1111/j.1464-5491.2011.03434.x.

Protocol Amendment Summary

Protocol Amendment Summary

Amendments to the YCMC Trial study protocol have been submitted and approved by the IRB. The following is a summary of the key changes made to the original version of the protocol that was approved and implemented. Based on the approved amendment documents, this summary was created on Dec. 22, 2023.

Protocol Title: Trial of a novel brief intervention on health behaviors for youth with chronic medical conditions
Protocol Number IRB-P00021649

Date: May 1, 2017
IRB Amendment approval Date: 4/28/2017
<u>Changes to the protocol:</u>

- To revise measurement battery assessment for baseline and 6 and 12 months follow up
- Inclusion of post intervention survey and new educational intervention materials
- Addition of a new cohort (inflammatory bowel disease patients in the gastroenterology clinic), and to increase the eligibility age bracket to include patients who are 18 years old at baseline.
- Revision of sample size for enrollment to 300 subjects at BCH, 450 overall
- Addition to allow participation at other study sites.
- Revision to the protocol smart form, protocol, and assent forms to account for changes noted above.

In relation to the above changes, inclusion criteria have been modified as the following:
We recruit patients from the Endocrinology, Rheumatology, and Gastroenterology Clinics at Boston Children's Hospital.
Eligibility Criteria: Inclusion for Patient:

- (Endocrinology Clinic) – Diagnosis of type 1 diabetes for at least one year
- (Rheumatology Clinic) – Diagnosis of a rheumatic condition (appropriate for intervention materials) for at least one year
- (Gastroenterology Clinic) – Diagnosis of Inflammatory Bowel Disease (Crohn's or Colitis) for at least one year
- Adolescents 14-18 years of age.
- Ability to read and understand English at a middle school level or greater.
- Consent to 6 month and 12 month follow up assessments.

Date: September 8, 2017
IRB Amendment approval Date: 8/2/2017
The Institutional Review Board has approved the amendment submitted 8/2/2017.
<u>Changes to the protocol:</u>

- To revise the protocol document and smart form to reflect administrative clarifications regarding eligibility criteria to clarify description of study requirements within the assent form.

Date: October 25, 2017
IRB Amendment approval Date: 10/25/2017
<u>Changes to the protocol:</u>
- To add finalized recruitment material -- educational intervention for IBD cohort document.

Date: October 16, 2020
IRB Amendment approval Date: 9/28/2020
<u>Changes to the protocol:</u>
Due to the COVID-19 Pandemic occurring globally, the project will follow up with this cohort of patients with an optional additional survey approximately 24 to 48 months post baseline. Due to the addition of this additional assessment, the following changes have been made to the protocol.
- Submission of survey questions for 24-48 month follow up.

Trial of a Novel Brief Intervention for Substance Use for Youth with Chronic Medical Conditions (YCMC)

Statistical Analysis Plan

Co-Principal Investigator: Elissa Weitzman, ScD, MSc

Co-Principal Investigator: Sharon Levy, MD, MPH

Boston Children's Hospital

1.0 INTRODUCTION

2.0 SUMMARY OF STUDY DESIGN AND PROCEDURES

2.1 Study Objectives

- **2.1.1 Primary Objectives**
- **2.1.2 Secondary Objectives**
- **2.1.3 Exploratory Objectives**

2.2 Study Design and Procedures

- **2.2.1 Study Design**
- **2.2.2 Study Assessments**
  - **2.2.2.1 Study Procedures Recruitment**
  - **2.2.2.2 Randomization**
- **2.2.3 Psycho-education**
- **2.2.4 Assessment Battery**
- **2.2.5 Measures (Reference: Protocol p. 9-18)**

2.3 Eligibility Criteria for Selection of Study Population

- **2.3.1 Inclusion Criteria**
- **2.3.2 Exclusion Criteria**

3.0 GENERAL ANALYSIS DEFINITIONS AND CONVENTIONS

3.1 Analysis Populations

- **3.1.1 Eligible Population**
- **3.1.2 Respondents Population**
- **3.1.3 Intent-to-Treat Population**

4.0 PARTICIPANT ENROLLMENT AND DISPOSITION

5.0 ANALYSIS OF PARTICIPANT BASELINE CHARACTERISTICS

6.0 OUTCOME ANALYSIS

6.1 Definition of Primary Outcome Measure

6.2 Analysis of the Primary Outcome Measure

6.3 Definition of the Secondary Outcome Measures

6.4 Analyses of the Secondary Outcome Measures

7.0 SAFETY

8.0 SIGNIFICANCE TESTING

9.0 SAMPLE SIZE AND POWER

10.0 SOFTWARE TO BE USED FOR ANALYSES

REFERENCE

11.0 UPDATES TO THE STATISTICAL ANALYSIS PLAN

LIST OF ABBREVIATIONS

BCH: Boston Children's Hospital

GAD-2: General Anxiety Disorder – 2 item scale

HTE: Heterogeneous Treatment Effects

IBD: Inflammatory Bowel Diseases

IRB: Institutional Review Board

JIA: Juvenile Idiopathic Arthritis

MTF: Monitoring the Future

NIDA: National Institute on Drug Abuse

PHQ-2: Patient Health Questionnaire-2

RCT: Randomized Controlled Clinical Trials

REDCap: Research Electronic Data Capture

S2BI: Screening to Brief Intervention

SBIRT: Screening, Brief intervention and Referral to Treatment

SLE: Systemic Lupus Erythematosus

T1D: Type 1 Diabetes Mellitus

TLFB: Timeline Follow-back Method Assessment

TGC: Take Good Care

YCMC: Youth with Chronic Medical Conditions

1.0 INTRODUCTION

The Statistical Analysis Plan (SAP) for the YCMC Study expands upon the statistical information presented in the protocol and describes all planned analyses for the primary, secondary, and exploratory outcome measures. The protocol has been submitted to and approved by the BCH IRB on 1/28/2016.

This SAP captures any updates to the protocol pertaining to data analysis planning.

**Table A: Planned Analysis**		
Content	Product	Analysis Approach
Explaining Disposition of Enrolled Patients	CONSORT Flow Chart	NA
Participant Baseline Characteristics for overall and by randomization	Table	Bivariate analysis
Analyses of Primary Outcome	Table	Regression analysis
Analyses of Secondary Outcome Measures	Table	Regression analysis
Analyses of the Exploratory Outcome Measures	Table / Figure	Regression analysis

2.0 SUMMARY OF STUDY DESIGN AND PROCEDURES

2.1 Study Objectives

2.1.1 Primary Objectives (Reference: Protocol: p.1, p.8, p.9)

The primary goal of this project is to develop a tailored screening, brief intervention and referral to treatment (SBIRT) model for youth with chronic medical conditions (YCMC) for delivery at point of care during a routine healthcare visit, through conducting a rigorous randomized control trial among adolescent patients with type-1 diabetes (T1D) or rheumatologic conditions.

Building onto an earlier phase of this project (Validating Adolescent SBIRT Measures), we will assess the validity and acceptability of a subset of brief substance-specific questions that are strongly associated with reports of current substance use behaviors and harms and that predict future substance use behaviors and harms.

Screening, tailored feedback and psychoeducation will all be delivered electronically. The brief intervention will target perceived risk of harm of a given substance, knowledge about substances, knowledge of disease specific and medication interactions, and intentions to use. These intermediary measures may serve as proxy markers that can be tracked and may predict substance use trajectories during a long term follow up period.

<u>Primary Outcome (Reference: protocol p. 20)</u>

Our primary outcome variables will be initiation and escalation of substance use. Initiation of use will be defined as any new use of substances at follow-up among those reporting no past-12-month use at

baseline. For alcohol use, escalation will be defined as, among drinkers, any increase in number of drinks (measured by TLFB) from baseline to follow-up. For marijuana use, escalation will be defined as, among users, any increase in days of smoking marijuana (measured by TLFB) from baseline to follow-up.

We will assess the magnitude and direction of the effects of hypothesized predictors on substance use. Specifically, we will examine the association between each factor and past year substance use, past 90 days use and number of drinks measured by TLFB. Multivariate logistic/linear regression, adjusting for demographic characteristics, will be performed. To evaluate the effect of intervention on substance use at 6- and 12-month follow-up, we will perform multivariate logistic regression adjusting for demographics, predictors of substance use and baseline knowledge. Separate models will be used to evaluate effects at 6- and 12-month.

Adjusted odds ratios will be computed for comparison between intervention and control groups. We will also evaluate how well the change of intermediary measures predicts the change of substances use. We will use multiple logistic regression incorporating all intermediary variables. Separate models will be used to assess the associations at 6- and 12-month.

We hypothesize that YCMC who receive the intervention will be less likely than their peers to initiate or escalate alcohol consumption 6- and 12-months post intervention. And, YCMC who receive the intervention will be less likely than their peers to initiate or escalate marijuana use 6- and 12-months post intervention.

<u>Secondary Outcomes (Reference: Protocol p. 21)</u>

The brief intervention will target perceived risk of harm of a given substance, knowledge about substances, knowledge of disease specific and medication interactions, and intentions to use. These intermediary measures may serve as proxy markers that can be tracked and may predict substance use trajectories during a long term follow up period.

We evaluate how well the change of intermediary measures predicts the change of substances use. We will use multiple logistic regression incorporating all intermediary variables. Separate models will be used to assess the associations at 6- and 12-month.

2.1.2 Secondary Objectives (Reference: Protocol p. 1, p.8, p.9)

The secondary goal is to assess the attitudes and knowledge of parents of these same adolescents, to explore associations between parent beliefs and adolescent substance use behavior. The measures include youth alcohol use, parent beliefs about their child’s alcohol use, and parental provision of alcohol guidance.

2.1.3 Exploratory Objectives (Reference: Protocol p. 1, p.8, p.9)

We will explore whether parent beliefs at baseline have any correlation with their child’s substance use behavior and/or attitudes and perceptions of risk.

2.2 Study Design and Procedures

2.2.1 Study Design (Reference: Protocol p. 6)

The proposed project is a randomized control trial to assess the efficacy of an SBIRT model tailored to youth with chronic medical conditions. This trial is being built into a larger, longitudinal cohort study to assess whether a set of brief, substance specific questions can accurately predict adolescent substance use outcomes when compared to more lengthy, criterion standard assessment questions, in cross-sectional and prospective analysis.

The first component of the project will consist of pre-testing an assessment battery and brief electronic intervention with a small sample of 14-17 year old youth. In the second component we will randomize consented eligible participants into intervention or control arms of the study and administer the 1) Baseline Assessment Battery and the 2) Brief Intervention OR Brief Control Information. In the third component we will follow up with all participants at 6 and then 12 months after study entry to reassess rates of substance use through the follow up assessment batteries.

At baseline, we will also attempt to enroll parents of all participants who are present at the time of enrollment. We will separately consent and administer a brief assessment battery to parents to assess their knowledge and attitudes of YCMC substance use, as well as opinions and attitudes regarding other health and risk behaviors.

2.2.2 Study Assessments

2.2.2.1 Study Procedures Recruitment (Reference: Protocol p. 7-8)

At baseline, a project Research Assistant (RA) will screen the Rheumatology and Endocrinology Clinic rosters (through PowerChart, Hyperspace, and/or Childrens360) to generate a roster of potentially eligible patients with upcoming outpatient appointments, including the Center for Ambulatory Transfusion (CAT-CR).The roster will be given to clinicians or the administrator within the department for approval if requested. The providers will review the list and let the RA(s) know which patients are okay to approach to invite to participate in the study.

The RA(s) will use the clinic roster to contact these eligible patients prior to their clinic appointment. The RA will first send a letter in the mail to participants briefly describing the study. The RA will then call patients closer to their scheduled clinic appointment to provide more details about the study, and to see if interested participants would potentially be able to arrive prior to their clinic appointment or stay after their appointment to complete the study.

If RAs are unable to contact participants prior to their clinic appointment through mail or phone, the RA will approach participants at the time of their appointment to ask about their interest in participating in a research project. The RA will approach participants at the time of their appointment to ask about their interest in participating in a research project. The RA will meet with interested and/or eligible patients in a private space in or near the clinic or the clinic waiting room to explain the purpose and details of the study to interested patients, and answer any questions. For all those who are interested in participation, the RA will ask the adolescent to complete some questions on a tablet computer to verify eligibility.

2.2.2.2 Randomization (Reference: Protocol p. 8)

For those who are eligible to participate in the study, the RA will then obtain assent and the participant will be randomized into either the intervention or control group. The RA will then administer the recruitment form, measurement battery, and either the test intervention or control intervention based on randomization.

2.2.3 Psychoeducation (Reference: Protocol p. 9)

The psychoeducation intervention includes visually appealing slides on alcohol use for YCMC. The slides contain quotes from research and user-friendly explanations. The intervention is self-administered on a tablet with privacy. Participants can manually advance or choose auto-play, with an average review time of 4 minutes.

Disease-specific versions of the intervention are developed. Each version addresses the health effects of alcohol use on disease processes and treatment for the respective conditions. The intervention aims to evoke emotional resonance and provide factual information about alcohol's risks for YCMC in the social and medical contexts.

2.2.4 Assessment Battery (Reference: Protocol p. 9-18)

Domains

The baseline assessment battery will be completed before starting the intervention/control. Both intervention and control groups will complete the baseline, 6-month, and 12-month assessments. Our assessment battery will cover:

1. Health information, including medical history.
2. Factors that may influence substance use, such as social support, family structure, extracurricular activities, and mental health.
3. Measures of alcohol, tobacco, marijuana, prescription drug, and other illicit drug use frequency and severity.
4. Intermediate measures related to substance use, such as attitudes, knowledge, risk tolerance, availability, and future use. Measures related to the participant's personality, such as self-efficacy and grit, will also be included.
5. Disease-specific questions about the burden of disease and disease management.

We will use previously validated tools selected for their clinical and research utility, simplicity, ease of administration, and interpretation. Participants will enter their questionnaire responses into a tablet computer at baseline. Follow-up assessments at 6 months and 12 months will be conducted over the phone or in person by the RA.

2.2.5 Measures (Reference: Protocol p. 9-18)

Health information: Past medical history may inform a participant's decisions to use or abstain from alcohol and other drug use.[1,2] Youth may use illicit substances to self-medicate, or may avoid substances because of specific health concerns. We will ask participants to report past medical history, and to rate their overall health. We will use previously validated questions to assess these domains. A question on past medical history about being treated with prescribed medications for anxiety, depression, or ADHD was created novel for this battery.

Other factors that may affect substance use: Multiple components of the day to day life of youth may predict a relationship to substance use patterns. Predictors of interest for this project include social support, parental monitoring and communication, sleep, and family medical history.

Current mental health status: The PHQ-2 is a self-administered, 2-item scale for detecting depression using DSM-IV diagnostic criteria[3]. The PHQ-2 has been studied extensively in large populations and

comes with considerable evidence for its validity. The PHQ-2 has been used extensively in primary care settings and adolescent populations. We will be administering the full PHQ-2 at baseline, 6 month follow up, and 12 month follow up.

The GAD-2 is a self-administered, 2 item scale to screen for anxiety disorders. It has high sensitivity and specificity for detecting generalized anxiety disorder and has been used in primary care settings[4]. We will be administering the full GAD-2 at baseline, 6 month follow up, and 12 month follow up.

Alcohol, tobacco, marijuana, and use of other substances: We will administer a series of questions about frequency of substance use and validate them against the criterion standard TLFB.

Substance use screen: We will administer the Screening to Brief Intervention (S2BI)[5] to ask about alcohol, tobacco, marijuana, prescription drug, and other substance use over the past 12 months. S2BI was developed at BCH with support from NIDA. In the original S2BI, questions on prescription drug use are asked only if patients answer yes to using alcohol, marijuana, or tobacco. In this study we will ask all participants about prescription drug use regardless of their use of other substances. Following the format of the S2BI, questions about other psychoactive substances including illegal drugs (such as cocaine or Ecstasy), inhalants (such as nitrous oxide), and herbs and synthetic drugs (such as salvia, "K2" or bath salts) will be asked only if participants answer yes to either alcohol, marijuana, or tobacco use.

Criterion standard for substance use disorder diagnosis: If participants answer no to past 12 month alcohol, marijuana, or tobacco use they will be asked a question about lifetime use of these substances. Questions about lifetime use were taken from the Computerized Diagnostic Interview Schedule for Children – 4 (DISC-IV).[6]

Alcohol safety screen: We will include questions about frequency and severity of alcohol use that will be used as safety flags. If a patient reports consuming 10 or more drinks containing alcohol in the past three months, or using sedatives in combination with alcohol, the RA will notify the primary clinician (See Adverse Event Criteria and Reporting Procedures)

Prescription medication misuse: Abuse of prescription medications among youth has risen dramatically in recent years. We will ask questions to assess if participants take medication as prescribed by their doctor. We will also assess medication diversion, with a specific interest in patients who are being prescribed stimulant medications. These questions were adapted from previous studies on prescription medication misuse among adolescents.[7]

Criterion standard substance use frequency measure: Each participant who reports any alcohol use will complete a 90-day Timeline Follow Back (TLFB) calendar that records frequency and quantity of alcohol use and frequency of marijuana use. The 90-day TLFB has been demonstrated to be both valid [8] and reliable [9] in adolescents. Participants will complete the TLFB on the computerized tablet following completion of the assessment battery. The TLFB is self-administered; participants will view an electronic calendar of the past 90 days and will input if they used marijuana on a given day and/or number of drinks consumed on a given day. If participants have not used marijuana or consumed a drink in the past 90 days, they will click through the calendar leaving the days blank. On the last screen they will be prompted to confirm that they did not select any days. The tool will be programmed to calculate total days of use and number of drinks.

Intermediary measures: We hypothesize that the following questions may serve as intermediary measures to predict long term substance use outcomes.

Time spent with alternative activities: Studies have shown that involvement in school based extra-curricular activities is generally related to less substance use among adolescents except in the case of athletic participation which has shown a positive relationship to substance use. We are interested in the number of hours participants spend per week engaged in different activities.[10]

Academic and extracurricular performance: Engagement, performance, and interest in academics and extracurricular activities, as well as disciplinary consequences may be indicators of current or future substance use. We will ask participants about personal consequences related to substance use. We are interested in consequences related to school such as academic performance, their commitment to school and extra-curricular activities, and how often they receive detention.

Perception of overscheduling: We will ask questions based on whether or not adolescents view themselves as overscheduled taken from the Measure of Perceived Over-Scheduling (MOPS) scale. We selected three items from the MOPS scale one from each factor measurement: pressure from external forces to participate in activities, feeling overwhelmed by participation, and preference for one's activities.[11]

Acceptability and interest in app/tools to monitor health behaviors/ Internet and social media use: We will ask questions about participant's willingness to use cellphone apps and tools to monitor health behaviors. This may provide information that can be used to inform development of health behavior interventions. Other factors that may also be of interest in informing interventions are the presence of alcohol and other drugs on social media websites and participant's use of the internet to search for information about alcohol or other drugs. These questions were created novel for this study.

Possible reasons to abstain from alcohol and marijuana use: We are interested in learning more about different reasons participants may choose to abstain from alcohol and marijuana use. We will ask participants to rate the importance of different statements as reasons to abstain from alcohol and marijuana. We will also assess whether perceived parental attitudes about smoking marijuana has an impact on marijuana use.

Knowledge about the impact of marijuana and alcohol on health: Brief interventions to prevent or reduce adolescent substance use often focuses on increasing youth knowledge of the harms associated with use, with the expectation that increased knowledge will lead to decreased use over time. We will ask novel questions to assess participants' knowledge on the effects of substance use.

Perceived risk of harm from substance use: In nationally representative surveys, perceived risk of harm related to substance use is strongly associated with marijuana use on a population level, though less is known about the predictive validity on an individual level. We will ask participants questions regarding perceived risk of harm of alcohol, marijuana, tobacco and prescription medications in order to describe associations between perceived risk of harm and substance use trajectories.

Perceived availability of alcohol and marijuana: Increased availability of alcohol has been associated with increased alcohol use and related problems in youth.[12] To look at perceived availability of substances and the correlation with substance use on an individual level, we will ask a question about how difficult it would be for participants to get alcohol and marijuana if they wanted some. These questions were taken from the Monitoring the Future study.

Perceived likelihood of future substance use: We will use questions from Monitoring the Future that ask about likelihood of future use validated in a longitudinal sample to determine whether this question is a good proxy measure of future use. We will ask participant about their likelihood of substance use three

months from when they are completing the assessment battery and analyze responses against reports of actual substance use collected longitudinally.

Receipt of healthcare advice about substances from a medical professional[13]**:** To determine adolescents' experience of physician screening we will whether a clinician has asked about substance use and willingness to talk to a clinician or learn more about how alcohol use can affect health. We will use questions that were piloted by the research team in a previous project (NIAAA number).

Confidence in refusal skills: We are interested in learning about participant's confidence in their ability to refuse substances from friends. An adolescent's peers can have an influence on their behavior or substance use.[14] We will include one novel question in our battery to assess participant's perceived self-efficacy.

Grit: Perseverance and passion for long term goals may predict substance use trajectories[15]. We will be administering the 8-Item Grit Scale, which is a two factor model that measures consistency of interest and perseverance of effort. The Grit Scale has been used in research settings and has been validated in both adult and adolescent populations[16]. In these populations it has been used to look at relationships in educational attainment, career changes, and predicted GPA.[16] We will include all 8 items from the Short Grit Scale in our assessment battery.

Future goals for after high school: Goals for the future may predict substance use by high school students. This has been studied in adjudicated adolescents, where positive future orientation was associated with lower levels of substance use.[17] There has been little research in goal setting and future orientation as a predictor for other populations of adolescents. We would like to include a section on future goals in our assessment battery to study it further as a predictor for substance use. We will ask participants whether they have plans for their future after high school.

Substance specific patient centered outcomes.

Problems and consequences associated with alcohol and marijuana use: Standard measures of consequences related to substance use typically mix all substances together, creating a composite scale. For this project, we will separately determine the most common consequences reported by adolescents associated with alcohol use and marijuana use. Using the Personal Experience Screening Questionnaire (PESQ)[18], we will ask participants substance specific outcome questions to determine whether alcohol and marijuana have different patterns of outcomes.

Sexual risk associated with substance use: Risky sexual contact is commonly associated with alcohol use.[19] Using questions adapted from the Youth Risk Behavior Survey[20], we will assess if a participant has ever engaged in sexual contact Specifically we are interested in whether there are different relationships and perceived concerns between alcohol and/or marijuana and unprotected sex.

Disease specific health questions

Disease management: We will ask a set of questions to learn more about current disease status and how patients manage their chronic medical conditions. Diabetes specific questions include use of an insulin pump, use of a glucose monitor, number of times he/she has had their hemoglobin A1c (HbA1c) checked by a doctor in the past 12 months, last HbA1c reading, and number of times they check their blood sugar daily. Rheumatologic condition specific questions include a pain scale and how often they have experienced morning stiffness in the past 30 days.

Disease severity: We will ask patients with type 1 diabetes about episodes of severe hyperglycemia and hypoglycemia and patients with rheumatological conditions about frequency of disease flares to learn more about their medical condition.

Intervention:

Those in the intervention arm will receive a computerized brief intervention composed of tailored feedback and psycho-education. Content in the intervention will focus on health promotion and will deliver positive messages about health.

The Co-PIs previously conducted a qualitative study (Validating NIAAA's Brief Screening Guide in Youth with Chronic Medical Conditions) among YCMC receiving care at BCH. The goal of the study was to understand how they navigate their chronic medical condition in general and in social situations, situations in which they have used or not used substances, and the quality of messages they have received about their health. The core domains this intervention will focus on were adapted from data collected during the qualitative study and a literature review on the topic. The domains were refined and then pilot tested with a sample of patients who participated in the aforementioned qualitative study.

Domains included in the intervention are 1) disclosure of disease to peers and resistance skills; 2) adolescent development; 3) medication adherence; 4) and disease specific knowledge. The topic area will include information on science or key findings in research, narratives from peers on their experiences, and actionable items or key messages that reinforce the topic. Quotes from youth with chronic medical conditions collected from the previous qualitative study were included as part of the narrative sections to provide participants with messages from peers.

Disclosure of disease to peers and resistance skills: Studies have shown that peer support from close friends had an impact on stressors such as adjusting to a chronic condition or coping with a difficult medical treatment in youth with chronic medical conditions.[21] We are also interested in including a section on refusal skills in out intervention. Findings suggest that resistance skills training may be especially effective before the onset of behavior as a primary prevention tool.[22] Youth who lack refusal skills are more likely to engage in alcohol use in early adolescence and this trend persists into later adolescence as well.[23] These two themes will be combined into a section about encouraging communication with peers if they have not already done so and increasing comfort in doing so. It also includes recommendations to use close friends in building resistance skills, having "sober buddies" and in supporting decisions not to use substances.

Adolescent Development: Adolescents growing up with a chronic medical condition face unique challenges and burdens compared to other adolescents. Findings from our qualitative study showed that many adolescents felt their condition and management of their health made them feel more responsible and mature compared to others their age. This section will focus on acknowledging that while YCMC may feel burdened by their condition there may be positive ways in which their condition has changed their life, including increased maturity and resilience.

Medication Adherence: A previous study by the Co-PIs found that high school youth who reported past year alcohol use were 1.79 times as likely to report they forgot to take their medications and 1.61 times as likely to report skipping their medications "always/ often/sometimes" over the past 30 days than youth who did not report past year alcohol use.[24] This section will provide information around medication adherence and the possible effects of substance use on adherence.

Disease Knowledge: A previous study by the Co-PIs also showed that knowledge about whether alcohol can interfere with medications or laboratory tests used to treat chronic conditions was low among YCMC. Alcohol use among youth with type 1 diabetes can pose serious health risks such as hypoglycemia.[25] A common theme in the qualitative work by the Co-PIs was a preference from adolescents for direct and hones information about the disease-specific risks of substance use without "any gray areas" or "sugarcoating" information[26]. The knowledge section will focus on presenting information about how alcohol is processed in the body and how this may impact chronic conditions or the effectiveness of the medications used to treat chronic conditions.

Control: Those in the control arm will receive information/brief advice around internet safety. Information for the internet safety was adapted from existing curriculum available through Netsmartz and KidsHealth.org. The core topic around internet safety will focus on how youth can protect their online presence and increase awareness of their "digital footprint". We will provide information on the importance of being safe about posting online, possible consequences of what posting personal information, and protecting online privacy.

Follow-up Assessment: We will follow up with the recruited sample 6 months and 12 months post baseline. We will administer an abbreviated Assessment Battery, either online or via phone, with a focus on changes in behavior since baseline to measure substance use and outcome trajectories over time. The assessment batteries administered at 6 and twelve months post baseline are attached to this protocol.

Parent Assessment Battery

We will recruit parents of participants enrolled in the trial to complete an assessment battery at baseline. We will ask parents questions to ascertain their opinions around a variety of health related issues and how they relate to their child with a chronic condition. Topics will include disease burden, mental health, care transition, knowledge and attitudes around substance use, social media use, and use of marijuana as medicine. Due to the a lack of studies that have looked specifically at parents of youth with chronic medical conditions and many of these topics, a number of our questions are novel, however questions in this survey have been pilot tested a well received in a related anonymous survey of parents whose children have chronic medical conditions (IRB-P00017232).

2.3 Eligibility Criteria for Selection of Study Population

2.3.1 Inclusion Criteria (Reference: Protocol p. 6)

14–17-year-old youth presenting for routine medical care in the Rheumatology clinic or Endocrinology clinic at BCH, with informed assent. To be eligible, participants in the Endocrinology clinic must have a diagnosis of type 1 diabetes for at least a year and participants in the Rheumatology clinic must have a diagnosis of a rheumatologic condition for at least a year. Eligible youth must also be between 14-17 years old at the time of enrollment and be able to read and understand English at a middle school level or greater. Participants must consent to participation in the study and consent to the 6 month and 12 month follow up assessments.

Parents whose children enrolled in the study, and who are able to read and understand English at a middle school level or greater will be eligible to participate in the parent study.

2.3.2 Exclusion Criteria (Reference: Protocol p. 7)

Patients who are medically or emotionally unstable or otherwise unable to provide assent at the time of their appointment as determined by their clinician or the research team, those who are unable to speak/read English at a middle school reading level, use a computer keyboard and/or complete an interviewer-assisted questionnaire will be excluded. Patients who do not consent to 6 month and 12 month re-assessment will also be excluded. Patients who are pregnant at baseline will be excluded from the study. If a participant enrolls in the study and becomes pregnant after baseline, they will not be excluded from the follow-up assessments.

Parents of enrolled participants who are not able to read and understand English at a middle school level or greater will be excluded. Parent ineligibility will not affect patient eligibility.

3.0 GENERAL ANALYSIS DEFINITIONS AND CONVENTIONS

3.1 Analysis Populations

3.1.1 Eligible Population (Reference: Protocol p. 7)

The eligible population consists of all patients who have been approached for the study.

3.1.2 Respondent Population (Reference: Protocol p. 7)

The respondent population consists of all participants who have provided informed consent during the recruitment process and have been screened for eligibility.

3.1.3 Intent-to-Treat Population (Reference: Protocol p. 7, p. 20)

The Intent-to-Treat (ITT) population consists of all participants who have provided informed consent at the initiation of the screening process, have been randomized, enrolled in the study, and have responded to the survey questions.

4.0 PARTICIPANT ENROLLMENT AND DISPOSITION (Reference: Protocol p. 20)

The number of respondents assessed for eligibility and participants who are enrolled, as well as those who have been included in the analytic sample, will be clarified in the CONSORT diagram. The distribution of participants' characteristics by inclusion/exclusion in the study will be assessed for any differences. Participant disposition will be summarized based on the number of participants who are retained in the study, complete the study, and the number of participants who withdrew from the study will be clarified in the CONSORT diagram.

5.0 ANALYSIS OF PARTICIPANT BASELINE CHARACTERISTICS (Reference: Protocol p. 20)

To assess baseline equivalence between intervention group and control group, we will compare distribution of demographic characteristics using $\chi 2$ test or Fisher exact test for categorical variables and t-test or Wilcoxon sign rank test for continuous variables. We will also evaluate equivalence among those lost to follow-up versus those retained by comparing demographics and outcomes among those lost and those with complete follow-up.

6.0 OUTCOME ANALYSIS

6.1 Definition of Primary Outcome Measure (Reference: Protocol p. 20)

We will assess the magnitude and direction of the effects of hypothesized predictors on substance use. Specifically, we will examine the association between each factor and past year substance use, past 90 days use and number of drinks measured by TLFB. Multivariate logistic/linear regression, adjusting for demographic characteristics, will be performed.

To evaluate the effect of intervention on substance use at 6- and 12-month follow-up, we will perform multivariate logistic regression adjusting for demographics, predictors of substance use and baseline knowledge. Separate models will be used to evaluate effects at 6- and 12-month. Adjusted odds ratios will be computed for comparison between intervention and control groups.

6.2 Analysis of the Primary Outcome Measure (Reference: Protocol p. 20)

All analyses will be conducted under the principle of intention to treat; all participants will be analyzed as members of the groups to which they were originally randomized to. We will examine the impact of the psychoeducation program on alcohol use, measured from baseline to twelve months, taking into account correlations within individuals, using multivariate mixed-effects models.

Our primary outcome variables will be initiation and escalation of substance use. Initiation of use will be defined as any new use of substances at follow-up among those reporting no past-12-month use at baseline. For alcohol use, escalation will be defined as, among drinkers, any increase in number of drinks (measured by TLFB) from baseline to follow-up. For marijuana use, escalation will be defined as, among users, any increase in days of smoking marijuana (measured by TLFB) from baseline to follow-up.

6.3 Definition of the Secondary Outcome Measures (Reference: Protocol p. 13- 21)

We will also evaluate how well the change of intermediary measures predicts the change of substances use. We will use multiple logistic regression incorporating all intermediary variables. Separate models will be used to assess the associations at 6- and 12-month. We hypothesize that the following questions may serve as intermediary measures to predict long term substance use outcomes.

Intermediary Measures

Knowledge about the impact of marijuana and alcohol on health

Brief interventions to prevent or reduce adolescent substance use often focuses on increasing youth knowledge of the harms associated with use, with the expectation that increased knowledge will lead to decreased use over time. We will ask novel questions to assess participants' knowledge on the effects of substance use.

Perceived risk of harm from substance use

In nationally representative surveys, perceived risk of harm related to substance use is strongly associated with marijuana use on a population level, though less is known about the predictive validity on an individual level. We will ask participants questions regarding perceived risk of harm of alcohol, marijuana, tobacco and prescription medications in order to describe associations between perceived risk of harm and substance use trajectories.

Perceived availability of alcohol and marijuana

Increased availability of alcohol has been associated with increased alcohol use and related problems in youth.[12] To look at perceived availability of substances and the correlation with substance use on an individual level, we will ask a question about how difficult it would be for participants to get alcohol and marijuana if they wanted some. These questions were taken from the Monitoring the Future study.

Perceived likelihood of future substance use

We will use questions from Monitoring the Future that ask about likelihood of future use validated in a longitudinal sample to determine whether this question is a good proxy measure of future use. We will ask participant about their likelihood of substance use three months from when they are completing the assessment battery and analyze responses against reports of actual substance use collected longitudinally.

Receipt of healthcare advice about substances from a medical professional

To determine adolescents' experience of physician screening we will whether a clinician has asked about substance use and willingness to talk to a clinician or learn more about how alcohol use can affect health. We will use questions that were piloted by the research team in a previous project.

Confidence in refusal skills

We are interested in learning about participant's confidence in their ability to refuse substances from friends. An adolescent's peers can have an influence on their behavior or substance use.[14] We will include one novel question in our battery to assess participant's perceived self-efficacy.

Grit

Perseverance and passion for long term goals may predict substance use trajectories[15]. We will be administering the 8-Item Grit Scale, which is a two factor model that measures consistency of interest and perseverance of effort. The Grit Scale has been used in research settings and has been validated in both adult and adolescent populations[16]. In these populations it has been used to look at relationships in educational attainment, career changes, and predicted populations.[16] We will include all 8 items from the Short Grit Scale in our assessment battery.

Future goals for after high school

Goals for the future may predict substance use by high school students. This has been studied in adjudicated adolescents, where positive future orientation was associated with lower levels of substance use.[17] There has been little research in goal setting and future orientation as a predictor for other populations of adolescents. We would like to include a section on future goals in our assessment battery to study it further as a predictor for substance use. We will ask participants whether they have plans for their future after high school.

<u>Substance Specific Patient Centered Outcomes.</u>

Problems and consequences associated with alcohol and marijuana use

Standard measures of consequences related to substance use typically mix all substances together, creating a composite scale. For this project, we will separately determine the most common consequences reported by adolescents associated with alcohol use and marijuana use. Using the Personal Experience Screening Questionnaire (PESQ)[18], we will ask participants substance specific outcome questions to determine whether alcohol and marijuana have different patterns of outcomes.

Sexual risk associated with substance use

Risky sexual contact is commonly associated with alcohol use.[19] Using questions adapted from the Youth Risk Behavior Survey[20], we will assess if a participant has ever engaged in sexual contact. Specifically, we are interested in whether there are different relationships and perceived concerns between alcohol and/or marijuana and unprotected sex.

6.4 Analyses of the Secondary Outcome Measures (Reference: Protocol p. 21)

We will also evaluate how well the change of intermediary measures predicts the change of substances use. We will use multiple logistic regression incorporating all intermediary variables. Separate models will be used to assess the associations at 6- and 12-month.

7.0 SAFETY (Reference: Protocol p. 19)

We do not anticipate any adverse events as a result of this study. The greatest risk is potential breach of confidentiality regarding sensitive information (e.g., use of drugs and alcohol). To protect against this possibility, we will use randomly generated study identifiers, created by a computer, in all surveys, transcripts and databases. Code keys that link the study identifier to the subject identity (medical record number, name, and telephone number) will be maintained throughout the study period. The keys will be stored and locked (password-protected) in a secure location.

Although not induced by study participation, serious substance use may be detected during completion of the assessment batteries. We will follow the same guidelines for assessment of risk related to substance use that has been used in protocols previously approved by the BCH institutional review board. If a participant responds "yes" to consuming 10 or more alcoholic drinks in one occasion within the past 3 months, using sedatives (such as benzodiazepine, opioids, barbiturates, or pain medication) in combination with alcohol, using stimulants (such as Adderall or Ritalin) in combination with alcohol, or using illegal drugs other than marijuana (such as cocaine, or Ecstasy) monthly, the RA will notify the primary clinician.

At baseline, the RA will locate the primary clinician in the clinic following completion of the assessment battery. The primary clinician will be able to re-interview the patient, as clinically warranted, and determine what further action should be taken. The RA will also notify the primary clinician if there is any indication or concerns about the participant's risk for imminent harm. We will ask the primary clinician to sign our Alcohol Safety Sheet (see attached) to indicate that he/she has been notified. We will tell participants that we cannot make absolute assurances or provide them with specific guidelines on parental notification.

We will explain that we will only inform their clinician and that the clinician will decide if a participant's parents should be notified based on the best clinical judgment at the time. We will assure participants that we will make every effort *to inform them before the fact* when breach of confidentiality is required, to include them in a discussion regarding the exact details that are to be released and to release as few details as possible to ensure the safety of the adolescent (or other person).

Although not induced by study participation, depressive symptoms may also be detected during the baseline study assessment through completion of the PHQ-2. If a participant scores 3 or greater on the PHQ-2, indicating a non-acute need for further assessment, the RA will inform the participant that we will be notifying his/her provider, who may follow up with them by phone, or at their next appointment. We will notify the participant's provider of the PHQ-2 score via secure e-mail by the end of the day. We will ask that the provider reply to our e-mail to confirm that he or she has been notified of the safety concern. The provider will then address the mental health safety concern according to standard clinical practice. If the RA does not receive confirmation of receipt from the clinician, the RA will follow up with a phone call and/or page to ensure that the message is received. Both Principal Investigators (Levy and Weitzman) will be alerted of all safety concerns via email.

At 6 and 12 months follow up safety concerns may also arise through completion of the assessment batteries. We will address follow up safety flags through secure e-mails to the participant's specialty care clinician. We will notify the clinician via secure e-mail if a participant has a PHQ-2 score of 3 or higher (indicating a non-acute need for further assessment), or if serious substance use is detected (high volume alcohol consumption or use of sedatives in combination), and request confirmation that the participant's clinician has received the notification and is using standard clinical protocol to determine appropriate follow-up and/or referrals. If we do not receive confirmation of receipt from the clinician, we will follow up with a phone call and/or page to ensure that the message is received. We will provide email notification to clinicians of any safety flags raised within 48 hours of survey completion.

We will provide participants with contact information for their primary and specialty care providers if they wish to talk to a health professional or obtain help regarding substance use and/or mental health. A text message or email will also be sent to participants via BCH's outlook email system with the names and telephone numbers of two resources outside of BCH they may call if they wish to talk to a professional or gain help regarding substance use and/or mental health. If the participant does not readily know their cell phone carrier, which is required for sending the text message via email, an external website will be utilized to identify the cell phone carrier. Participant will also be provided verbally with the contact numbers for these resources upon request, or if they do not wish to receive a text message, but would still like the information.

It is also possible, but unlikely, that some participants could become upset by items in the measurement battery or information presented in the intervention or control arms. We include statements at the start that participants may skip questions that they are uncomfortable answering, that they may stop the study at any time, and that they may speak with the RA after completing the measurement if they have any questions or concerns about the questions asked or the material presented.

Given the nature of the study, we anticipate few if any adverse events. If we determine during the course of the study that an adolescent or someone else is at serious risk of harm, we will notify their provider. We will inform adolescents of this before we ask them to assent to participation in the study. We do not anticipate any serious adverse events, such as medical complications or deaths, as a result of this project.

8.0 SIGNIFICANCE TESTING

Statistical significance will be considered at $p < .05$ for each analysis.

9.0 SAMPLE SIZE AND POWER (Reference: Protocol p. 21)

We will recruit a convenience sample of 450 adolescent patients (n=450) and expect 300 of them will complete the entire study. With total sample size of 300 youth, there is adequate power (.80) to detect

significant effects ($p<.05$) for adjusted odds ratios of ≥ 2.0, defined as the likelihood that control versus intervention participants initiate or escalate their substance use behaviors from baseline to follow-up, across prevalence levels in the intervention group that range from .20 to .55.

10.0 SOFTWARE TO BE USED FOR ANALYSES

All data management and analyses will use SAS Version 9.4 software.

Reference:

1. Surís JC, Michaud PA, Akre C, Sawyer SM. Health risk behaviors in adolescents with chronic conditions. *Pediatrics*. 2008;122(5):e1113-8. doi:10.1542/peds.2008-1479

2. Increased Prevalence of Risk Behaviors Reported by Chronically Ill Adolescents. *NEJM Journal Watch*. 2008;2008. doi:10.1056/PA200812100000003

3. Kroenke K, Spitzer RL, Williams JBW. The Patient Health Questionnaire-2: validity of a two-item depression screener. *Med Care*. 2003;41(11):1284-1292. doi:10.1097/01.MLR.0000093487.78664.3C

4. Skapinakis P. The 2-item Generalized Anxiety Disorder scale had high sensitivity and specificity for detecting GAD in primary care. *Evid Based Med*. 2007;12(5):149. doi:10.1136/ebm.12.5.149

5. Levy S, Weiss R, Sherritt L, et al. An Electronic Screen for Triaging Adolescent Substance Use by Risk Levels. *JAMA Pediatr*. 2014;168(9):822-828. doi:10.1001/jamapediatrics.2014.774

6. Schwab-Stone ME, Shaffer D, Dulcan MK, et al. Criterion validity of the NIMH Diagnostic Interview Schedule for Children Version 2.3 (DISC-2.3). *J Am Acad Child Adolesc Psychiatry*. 1996;35(7):878-888. doi:10.1097/00004583-199607000-00013

7. McCabe SE, Teter CJ, Boyd CJ. Medical use, illicit use and diversion of prescription stimulant medication. *J Psychoactive Drugs*. 2006;38(1):43-56. doi:10.1080/02791072.2006.10399827

8. Knight JR, Sherritt L, Shrier LA, Harris SK, Chang G. Validity of the CRAFFT substance abuse screening test among adolescent clinic patients. *Arch Pediatr Adolesc Med*. 2002;156(6):607-614.

9. Levy S, Sherritt L, Harris SK, et al. Test-retest reliability of adolescents' self-report of substance use. *Alcohol Clin Exp Res*. 2004;28(8):1236-1241.

10. Farb AF, Matjasko JL. Recent advances in research on school-based extracurricular activities and adolescent development. *Developmental Review*. 2012;32(1):1-48. doi:10.1016/j.dr.2011.10.001

11. Pollock A. The Development of the Measure of Perceived Overscheduling (MOPS). *PCOM Psychology Dissertations*. 2010:Paper 172.

12. Treno AJ, Ponicki WR, Remer LG, Gruenewald PJ. Alcohol outlets, youth drinking, and self-reported ease of access to alcohol: a constraints and opportunities approach. *Alcohol Clin Exp Res*. 2008;32(8):1372-1379. doi:10.1111/j.1530-0277.2008.00708.x

13. Weitzman ER, Nelson TF. College student binge drinking and the "prevention paradox": implications for prevention and harm reduction. *J Drug Educ*. 2004;34(3):247-265.

14. Allen JP, Chango J, Szwedo D, Schad M, Marston E. Predictors of susceptibility to peer influence regarding substance use in adolescence. *Child Dev*. 83(1):337-350. doi:10.1111/j.1467-8624.2011.01682.x

15. Duckworth AL, Peterson C, Matthews MD, Kelly DR. Grit: perseverance and passion for long-term goals. *J Pers Soc Psychol*. 2007;92(6):1087-1101. doi:10.1037/0022-3514.92.6.1087

16. Duckworth AL, Quinn PD. Development and validation of the short grit scale (grit-s). *J Pers Assess*. 2009;91(2):166-174. doi:10.1080/00223890802634290

17. Robbins RN, Bryan A. Relationships Between Future Orientation, Impulsive Sensation Seeking, and Risk Behavior Among Adjudicated Adolescents. *J Adolesc Res*. 2004;19(4):428-445. doi:10.1177/0743558403258860

18. Winters KC. Development of an adolescent alcohol and other drug abuse screening scale: Personal experience screening questionnaire. *Addictive Behaviors*. 1992;17:479-490.

19. Cooper ML. Alcohol use and risky sexual behavior among college students and youth: evaluating the evidence. *J Stud Alcohol Suppl*. 2002;(14):101-117.

20. Center for Disease Control and Prevention. *2015 State and Local Youth Risk Behavior Survey*.; 2015.

21. La Greca AM, Bearman KJ, Moore H. Peer relations of youth with pediatric conditions and health risks: promoting social support and healthy lifestyles. *J Dev Behav Pediatr*. 2002;23(4):271-280.

22. Komro KA, Perry CL, Williams CL, Stigler MH, Farbakhsh K, Veblen-Mortenson S. How did Project Northland reduce alcohol use among young adolescents? Analysis of mediating variables. *Health Educ Res*. 2001;16(1):59-70.

23. Scheier LM, Botvin GJ, Diaz T, Griffin KW. Social skills, competence, and drug refusal efficacy as predictors of adolescent alcohol use. *J Drug Educ*. 1999;29(3):251-278.

24. Weitzman ER, Ziemnik RE, Huang Q, Levy S. Alcohol and Marijuana Use and Risks for Treatment Non-Adherence Among Medically Vulnerable Youth. *Pediatrics*. (in press).

25. Canadian Diabetes Association. *Alcohol + Diabetes Frequency Asked Questions for Healthcare Professionals Nutrition Guidelines Implementation Subcommittee Question*.; 2006.

26. Weitzman ER, Salimian PK, Rabinow L, Levy S. Shaped By My Disease: Perspectives on Substance Use Shared by Youth with Chronic Medical Conditions. In: *National Pediatric Academic Societies Meeting*. ; 2015.

11.0 UPDATES TO STATISTICAL ANALYSIS

Updates in relation to 6.0 OUTCOME ANALYSIS

Updated Date: 11/15/2016

The randomization checklist includes stratification by age and gender at each site. Within each stratum, treatment assignments will be randomly generated and assigned.

Updates in relation to 6.0 OUTCOME ANALYSIS

Updated Date: 6/27/2019

Defining Outcomes

Primary Outcome(s): Past 3-month alcohol use measured by a Brief Frequency variable for alcohol use.

Updates in relation to 6.0 OUTCOME ANALYSIS

Updated Date: 12/16/2020

Data Analysis Approach

The impact of the psychoeducation program on alcohol use, measured from baseline to twelve months, taking into account correlations within individuals, using multivariate mixed-effects models adjusting for baseline characteristics.

Updates in relation to 6.0 OUTCOME ANALYSIS

Updated Date: 09/28/2023

Post-hoc Exploratory Analysis for Heterogeneous Treatment Effects (HTE)

The aim of this post-hoc analysis is to investigate the exploratory research question: Is there heterogeneity in the effect of the psychoeducation intervention, (Take Good Care: TGC), among YCMC with baseline high-risk (binge/risky alcohol use) and low-risk baseline alcohol use status, regarding the changes in the number of drinking days in the past 3 months over time?

We hypothesize that there would be differences in the intervention effect between youths with baseline high and low-risk alcohol use.

Definition of baseline high-risk alcohol use

The baseline high-risk alcohol use is defined as self-reporting of any past 12-month alcohol-related injury, ER visit, vomiting, or past 3-month excessive (“binge”) alcohol use at baseline.
