# Supplementary material for: Disease-Tailored Brief Intervention for Alcohol Use Among Youths With Chronic Medical Conditions: A Secondary Analysis of a Randomized Clinical Trial
Source: JAMA Netw Open. 2024 Jul 10;7(7):e2419858. doi: 10.1001/jamanetworkopen.2024.19858 (PMC11238030; doi:10.1001/jamanetworkopen.2024.19858)
Supplement: Supplement 2. — eTable 1. Alcohol Health Risk Knowledge Survey Questions eTable 2. Baseline Characteristics by 12-Month Follow-Up Participation eTable 3. Observed Outcomes at All Assessment Occasions eTable 4. Sensitivity Analyses eTable 5. Intervention Effect on Alcohol Use Initiation Among Youths Reporting No Lifetime Alcohol Use at Baseline eTable 6. Cannabis and Nicotine Use at All Assessment Occasions eTable 7. Intervention Effects Stratified by Baseline High-Risk Alcohol Use Behavior, Adjusting for Cannabis and Nicotine Use eTable 8. Cannabis and Nicotine Use by Intervention Effects and High-Risk Alcohol Use Behavior at Baseline eMethods eReferences [file jamanetwopen-e2419858-s002.pdf]

Supplementary Online Content

Weitzman ER, Minegishi M, Dedeoglu F, et al. Disease-tailored brief intervention for alcohol use among youths with chronic medical conditions: a secondary analysis of a randomized clinical trial. *JAMA Netw Open*. 2024;7(7):e2419858. doi:10.1001/jamanetworkopen.2024.19858

eTable 1. Alcohol Health Risk Knowledge Survey Questions
eTable 2. Baseline Characteristics by 12-month Follow-up Participation
eTable 3. Observed Outcomes at All Assessment Occasions
eTable 4. Sensitivity Analyses
eTable 5. Intervention Effect on Alcohol Use Initiation Among the Youth Reporting No Lifetime Alcohol Use at Baseline
eTable 6. Cannabis and Nicotine Use at All Assessment Occasions
eTable 7. Intervention Effects Stratified by Baseline High-Risk Alcohol Use Behavior, Adjusting for Cannabis and Nicotine Use
eTable 8. Cannabis and Nicotine Use by Intervention Effects and High-Risk Alcohol Use Behavior at Baseline
eMethods
eReference

This supplementary material has been provided by the authors to give readers additional information about their work.

© 2024 Weitzman ER et al. JAMA Network Open.

eTable 1. Alcohol Health Risk Knowledge Survey Questions

Clinic	Survey Questions	Answer Key
**Common Questions**	Alcohol is a depressant and can make feelings of sadness and anxiety worse	TRUE
	Drinking alcohol can make managing my [condition] even more difficult	TRUE
**Endocrinology Clinic Survey Questions**	Drinking alcohol causes the liver to release sugar into the bloodstream	TRUE
	Drinking alcohol can cause me to have low blood sugar (hypoglycemia) while I'm asleep	TRUE
	It can be difficult to predict what my blood sugar will do with alcohol in my system	TRUE
	It is easy to tell the difference between being drunk and having low blood sugar	FALSE
	Drinking alcohol can prevent glucagon from doing its full job	TRUE
**Rheumatology Clinic Survey Questions**	Alcohol temporarily damages liver cells	TRUE
	The liver works to process medications before it processes alcohol	FALSE
	When alcohol is in the body, rheumatic medications can cause harm to the liver	TRUE
	Alcohol can make it difficult for my doctor to know how I am tolerating my medications	TRUE
	After drinking alcohol, taking medications like Advil or Tylenol can be dangerous to the kidneys, because of dehydration	TRUE
**Gastroenterology Clinic Survey Questions**	Alcohol temporarily damages liver cells	TRUE
	Alcohol can alter the GI tract's bacterial composition	TRUE
	Drinking alcohol can let bacteria that are meant to be inside your GI tract have access to other parts of your body, causing inflammation	TRUE
	Drinking alcohol can reduce your risk of having a flare-up	FALSE
	IBD symptoms can worsen when you drink alcohol	TRUE
	It is safe to drink alcohol while on medication for IBD	FALSE

© 2024 Weitzman ER et al. JAMA Network Open.

eTable 2. Baseline Characteristics by 12-month Follow-up Participation[a]

Characteristic	**Overall**, N = 451	**Participated**, n = 410	**Not Participated at 12-month Follow-up**, n = 41	**p-value**[b]
**Age at the Baseline, Mean (SD)**	16.0 (1.4)	16.0 (1.4)	16.2 (1.4)	0.39
**Education Level**				0.14
Middle or High school	387 (85.8)	355 (86.6)	32 (78.0)	
College or Vocational/Other	64 (14.2)	55 (13.4)	9 (22.0)	
**Clinic**[c]				0.26
Endocrinology	212 (47.0)	188 (45.9)	24 (58.5)	
Rheumatology	114 (25.3)	107 (26.1)	7 (17.1)	
Gastroenterology	125 (27.7)	115 (28.0)	10 (24.4)	
**Gender** [d]				0.35
Male	217 (48.1)	193 (47.1)	24 (58.5)	
Female	229 (50.8)	212 (51.7)	17 (41.5)	
Other/Unknown	5 (1.1)	5 (1.2)	0 (0.0)	
**Randomized Group**				0.91
Intervention	224 (49.7)	204 (49.8)	20 (48.8)	
TAU	227 (50.3)	206 (50.2)	21 (51.2)	
**Race**[d]				0.37
Asian	8 (1.8)	8 (2.0)	0 (0.0)	
Black	18 (4.0)	14 (3.4)	4 (9.8)	
White	383 (84.9)	349 (85.1)	34 (82.9)	
Multiple or Other [e]	40 (8.9)	37 (9.0)	3 (7.3)	
Prefer not to answer	2 (0.4)	2 (0.5)	0 (0.0)	
**Ethnicity**[d]				0.30
Hispanic or Latino	39 (8.6)	33 (8.0)	6 (14.6)	
Non-Hispanic or Non-Latino	408 (90.5)	373 (91.0)	35 (85.4)	
Prefer not to answer	4 (0.9)	4 (1.0)	0 (0.0)	
**Parental Education Level**				0.047
Less than college	108 (23.9)	93 (22.7)	15 (36.6)	
College or higher	343 (76.1)	317 (77.3)	26 (63.4)	
**Alcohol Use**				
In past 12-month, any [f]	108 (23.9)	96 (23.4)	12 (29.3)	0.092
In past 3-month, any	90 (20.0)	79 (19.3)	11 (26.8)	0.10
Frequency [g], Mean (SD)	1.0 (3.0)	0.8 (2.5)	2.4 (5.9)	0.13
(Missing)	4	3	1	
Heavy episodic or 'binge' drinking, any [h]	39 (8.6)	32 (7.8)	7 (17.1)	0.065
**Alcohol-related Harms** (blackout/injury /ED visit/vomiting)[i], any	33 (7.3)	26 (6.3)	7 (17.1)	0.012
**High-Risk Alcohol Behavior** (binge/blackout/injury/injury/ED visit/vomiting)[j], any	52 (11.5)	42 (10.2)	10 (24.4)	0.017
**Alcohol Health Risk Knowledge (% correct)[k], Mean (SD)**	64.4 (25.9)	64.9 (25.6)	60.1 (28.8)	0.36
**Alcohol Risk Intolerance**[l]				0.48
Any number of drinks per occasion is risky	176 (39.0)	162 (39.5)	14 (34.1)	
>1 drink per occasion is risky	44 (9.8)	42 (10.2)	2 (4.9)	
>2 drinks per occasion is risky	83 (18.4)	77 (18.8)	6 (14.6)	
>3 drinks per occasion is risky	59 (13.1)	52 (12.7)	7 (17.1)	
>4 drinks per occasion is risky	33 (7.3)	29 (7.1)	4 (9.8)	
>5 drinks per occasion is risky/alcohol not risky	50 (11.1)	43 (10.5)	7 (17.1)	

© 2024 Weitzman ER et al. JAMA Network Open.

Characteristic	**Overall**, N = 451	**Participated**, n = 410	**Not Participated at 12-month Follow-up**, n = 41	p-value[b]
(Missing)	6 (1.3)	5 (1.2)	1 (2.4)	
**Alcohol Risk Intolerance (Likert scaled)[l], Mean (SD)**	3.3 (1.7)	3.3 (1.7)	2.9 (1.9)	0.15
(Missing)	6	5	1	

Abbreviations. ED, Emergency Department; TAU, Treatment As Usual.

a. Mean (SD); No. (Column %)
b. Wilcoxon rank sum test; Pearson's Chi-squared test; Fisher's exact test
c. The participants with type 1 diabetes were recruited from the endocrinology clinic, the participants with juvenile idiopathic arthritis (JIA) or systemic lupus erythematosus (SLE) were recruited from the rheumatology clinic, and those with inflammatory bowel disease (IBD) (i.e., diagnosed with ulcerative colitis or Crohn's disease) were seen at the gastroenterology clinic.
d. Self-reported.
e. Created due to the small sample size; includes participants who selected more than 1 race or any American Indian or Alaska Native, Hawaiian or Other Pacific Islander, or Other race.
f. For the survey question ("In the past 12 months, how many times have you used Alcohol?), the response [Never / Once or twice / Monthly /Weekly or more] was dichotomized as "any vs. never." Among the randomized, 2 youths did not contribute to the response.
g. For the survey question ("In the last three months, how many days did you have a drink containing alcohol?"), the responses were assessed among 447, 4 youths did not contribute to the response. Those screened as negative for the past 12 months of alcohol use were assigned 0 and were included in the analysis.
h. The criteria for heavy episodic or 'binge' drinking were defined as follows: female youths aged 14-17 years having 3 drinks containing alcohol on 1 occasion; female youths aged 18 years or male youths or youths of other genders aged 14-15 years having 4 drinks containing alcohol on 1 occasion, and male youths or youths of other genders aged 16-18 years having 5 drinks containing alcohol on 1 occasion.
i. Alcohol-related harms (blackout, injury, ED visit, or vomiting) were assessed with the following questions: "In the past 12 months, how often did you have blackouts while drinking alcohol when you couldn't remember afterward what had happened?", "How often have you been injured during or after drinking alcohol?", "How often have you gone to the emergency room because of problems related to your alcohol use?", and "How often have you vomited or thrown up from alcohol use?". The responses ("Never," "Once or twice," "Sometimes," and "Often") were dichotomized as any vs. never for the analysis.
j. High-risk alcohol use behavior was defined as self-reporting any of the following at baseline: heavy episodic or 'binge' drinking in the past 3 months or alcohol-related harms (blackout/injury/ER visit/vomiting) in the past 12 months.
k. Alcohol health risk knowledge scaled score indicates the percentage of questions answered correctly out of 100.
l. For the survey question ("How many drinks on one occasion would you consider risky or dangerous drinking for yourself?"), the 6-point Likert scale was revers-coded such that the higher score indicates a higher intolerance of alcohol risk. For example, 5 indicates that any number of drinks per occasion is risky; 4, >1 drink per occasion is risky; 3, >2 drinks per occasion is risky; 2, >3 drinks per occasion is risky; 1, >4 drinks per occasion is risky; and Scale 0, >5 drinks per occasion is risky/alcohol not risky.

© 2024 Weitzman ER et al. JAMA Network Open.

eTable 3. Observed Outcomes at All Assessment Occasions[a]

		Baseline		6-month Follow-up		12-month Follow-up	
**Group**	**Characteristic**	**Intervention**	**TAU**	**Intervention**	**TAU**	**Intervention**	**TAU**
		**n=26**	**n=26**	**n=26**	**n=26**	**n=26**	**n=26**
**High-risk Use at Baseline (n=52)[b]**	Alcohol Use						
	Frequency in Past 3 months						
	Mean (SD)	6.3 (4.6)	5.5 (4.9)	5.8 (4.9)	5.6 (5.4)	4.9 (4.3)	9.0 (5.8)
	(Missing)	2	0	1	1	3	7
	Alcohol Health Risk Knowledge (% correct)[c]						
	Mean (SD)	68.1 (22.2)	65.2 (25.3)	72.4 (23.6)	75.4 (22.2)	77.5 (23.4)	82.2 (17.8)
	(Missing)			1	1	3	7
	Alcohol Risk Intolerance (Likert scaled)[d]						
	Mean (SD)	1.4 (1.7)	1.0 (1.3)	1.7 (1.7)	1.6 (1.7)	1.8 (1.7)	1.4 (1.3)
	(Missing)			2	1	3	7
	Alcohol Risk Intolerance[d]						
	Any number of drinks per occasion is risky	3 (11.5)	1 (3.8)	3 (12.5)	3 (12.0)	2 (8.7)	1 (5.3)
	>1 drink per occasion is risky	1 (3.8)	1 (3.8)	0 (0.0)	0 (0.0)	2 (8.7)	0 (0.0)
	>2 drinks per occasion is risky	2 (7.7)	1 (3.8)	4 (16.7)	4 (16.0)	3 (13.0)	2 (10.5)
	>3 drinks per occasion is risky	4 (15.4)	4 (15.4)	6 (25.0)	4 (16.0)	5 (21.7)	5 (26.3)
	>4 drinks per occasion is risky	4 (15.4)	7 (26.9)	2 (8.3)	4 (16.0)	4 (17.4)	5 (26.3)
	>5 drinks per occasion is risky/alcohol not risky	12 (46.2)	12 (46.2)	9 (37.5)	10 (40.0)	7 (30.4)	6 (31.6)
	(Missing)			2	1	3	7
		n=198	n=201	n=198	n=201	n=198	n=201
**No or Low-risk Use at Baseline (n=399)**	Alcohol Use						
	Frequency in Past 3 months						
	Mean (SD)	0.3 (1.4)	0.2 (1.1)	0.8 (2.1)	0.6 (1.9)	1.6 (3.8)	0.8 (2.3)
	(Missing)	2	0	13	16	20	17
	Alcohol Health Risk Knowledge (% correct) [c]						
	Mean (SD)	63.7 (26.9)	64.6 (25.6)	76.7 (24.6)	68.1 (26.5)	79.3 (23.5)	70.5 (26.5)
	(Missing)			9	14	17	14
	Alcohol Risk Intolerance (Likert scaled)[d]						
	Mean (SD)	3.5 (1.7)	3.6 (1.5)	3.8 (1.5)	3.6 (1.5)	3.5 (1.6)	3.6 (1.6)
	(Missing)	5	1	11	15	21	16
	Alcohol Risk Intolerance[d]						
	Any number of drinks per occasion is risky	86 (44.6)	86 (43.0)	91 (48.7)	76 (40.9)	73 (41.2)	86 (46.5)

© 2024 Weitzman ER et al. JAMA Network Open.

		Baseline		6-month Follow-up		12-month Follow-up	
**Group**	**Characteristic**	**Intervention**	**TAU**	**Intervention**	**TAU**	**Intervention**	**TAU**
	>1 drink per occasion is risky	16 (8.3)	26 (13.0)	32 (17.1)	28 (15.1)	28 (15.8)	19 (10.3)
	>2 drinks per occasion is risky	37 (19.2)	43 (21.5)	21 (11.2)	41 (22.0)	28 (15.8)	33 (17.8)
	>3 drinks per occasion is risky	28 (14.5)	23 (11.5)	26 (13.9)	23 (12.4)	26 (14.7)	30 (16.2)
	>4 drinks per occasion is risky	8 (4.1)	14 (7.0)	5 (2.7)	8 (4.3)	12 (6.8)	6 (3.2)
	>5 drinks per occasion is risky/alcohol not risky	18 (9.3)	8 (4.0)	12 (6.4)	10 (5.4)	10 (5.6)	11 (5.9)
	(Missing)	5	1	11	15	21	16

Abbreviations TAU, Treatment as Usual; ED, Emergency Department

a. No (Column %), or Mean (SD).
b. High-risk alcohol use behavior was defined as self-reporting any of the following at baseline: heavy episodic or 'binge' drinking in the past 3 months or alcohol-related harms (blackout/injury/ED visit/vomiting) in the past 12 months.
c. Alcohol health risk knowledge scaled score indicates the percentage of questions answered correctly out of 100.
d. For the survey question ("How many drinks on one occasion would you consider risky or dangerous drinking for yourself?"), the 6-point Likert scale was revers-coded such that the higher score indicates a higher intolerance of alcohol risk. For example, 5 indicates that any number of drinks per occasion is risky; 4, >1 drink per occasion is risky; 3, >2 drinks per occasion is risky; 2, >3 drinks per occasion is risky; 1, >4 drinks per occasion is risky; and Scale 0, >5 drinks per occasion is risky/alcohol not risky.

© 2024 Weitzman ER et al. JAMA Network Open.

eTable 4. Sensitivity Analyses

Outcome	Intervention Effects [a]		
**Alcohol Use Frequency in Past 3 months**	**Exponentiated (ARRR)**	**(95% CI)**	**p-value**
**High-Risk Use at Baseline (n=52)[b] (Time x Intervention)**	0.64	(0.42 to 0.96)	0.03
**No or Low-risk Use at Baseline (n=399) (Time x Intervention)**	1.17	(0.84 to 1.63)	0.35

Abbreviation: ARRR, adjusted relative rate ratio; ED, Emergency Department

a. Sensitivity analyses for the primary outcomes were conducted by repeating the following outcome models using 100 imputed datasets created by Multiple Imputation (MI) with the Fully Conditional Specification (FCS) Regression and Predictive Mean Matching Method (REGPMM), assuming missing not at random (MNAR). All the models include interaction between time, intervention and high vs. no or low-risk use groups and adjusted for the baseline demographics, including parental education (less than college vs. college or higher) and participants' grades (middle/high school vs. after high school [college or vocational school]).
b. High-risk alcohol use behavior was defined as self-reporting any of the following at baseline: heavy episodic or 'binge' drinking in the past 3 months or alcohol-related harms (blackout/injury/ED visit/vomiting) in the past 12 months.

© 2024 Weitzman ER et al. JAMA Network Open.

eTable 5. Intervention Effect on Alcohol Use Initiation Among the Youth Reporting No Lifetime Alcohol Use at Baseline[a]

				Alcohol Use Initiation			
		Alcohol Use		Unadjusted		Adjusted[b]	
**Predictor**	**N**	**Never**, N = 214	**Initiated**, N = 47	**OR (95% CI)**	**p-value**	**AOR (95% CI)**	**p-value**
**Intervention**	261				0.55		0.45
TAU		115 (53.7)	23 (48.9)	1.00		1.00	
Intervention		99 (46.3)	24 (51.1)	1.21 (0.64 to 2.29)		1.28 (0.67 to 2.44)	

Abbreviation. OR, Odds Ratio; AOR, Adjusted Odds Ratio; TAU, Treatment as Usual.

a. Logistic regression assessed the intervention effects on alcohol initiation as outcomes among the youth who reported no lifetime use of alcohol at baseline. Alcohol initiation was defined as reporting alcohol use at either a 6- or 12-month follow-up survey. Among 290 participants with no lifetime alcohol use at baseline, a total of 29 participants were excluded due to missing data on alcohol use (n=4), non-participation in both follow-up surveys, or missing one of the follow-up surveys while reporting no alcohol use in the other (n=25).

b. The adjusted models controlled for parental education (less than college vs. college or higher) and participants' grades (middle/high school vs. after high school [college or vocational school]).

© 2024 Weitzman ER et al. JAMA Network Open.

eTable 6. Cannabis and Nicotine Use at All Assessment Occasions[a]

		Baseline		6-month Follow-up		12-month Follow-up	
**Group**	**Characteristic, No (%)**	**Intervention**	**TAU**	**Intervention**	**TAU**	**Intervention**	**TAU**
		**n=26**	**n=26**	**n=26**	**n=26**	**n=26**	**n=26**
**High-risk Alcohol Use at Baseline (n=52)[b]**	Cannabis Use Frequency in Past 3 months [c]						
	Mean (SD)	7.8 (17.1)	5.8 (13.8)	8.2 (19.8)	9.5 (21.4)	9.8 (19.3)	8.4 (14.5)
	(Missing)	2	0	1	1	3	7
	Cannabis Use in Past 6 months, any [d]						
		17 (68.0)	13 (50.0)	14 (56.0)	13 (52.0)	16 (69.6)	11 (57.9)
	(Missing)	1	0	1	1	3	7
	Nicotine Use in Past 6 months, any [e]						
		15 (57.7)	10 (38.5)	15 (60.0)	11 (44.0)	13 (59.1)	11 (61.1)
	(Missing)			1	1	4	8
		**n=198**	**n=201**	**n=198**	**n=201**	**n=198**	**n=201**
**No or Low-risk Alcohol Use at Baseline (n=399)**	Cannabis Use Frequency in Past 3 months [c]						
	Mean (SD)	2.1 (9.7)	0.9 (6.4)	2.9 (11.7)	1.6 (8.2)	5.2 (16.1)	1.9 (9.3)
	(Missing)	1	1	12	16	20	18
	Cannabis Use in Past 6 months, any [d]						
		28 (14.2)	14 (7.0)	39 (21.0)	21 (11.4)	44 (24.7)	30 (16.4)
	(Missing)	1	0	12	16	20	18
	Nicotine Use in Past 6 months, any [e]						
		25 (12.8)	16 (8.0)	33 (17.6)	22 (11.8)	34 (19.1)	24 (13.0)
	(Missing)	2	1	11	15	20	17

Abbreviations TAU, Treatment as Usual.

a. No (Column %), or Mean (SD).
b. High-risk alcohol use behavior was defined as self-reporting any of the following at baseline: heavy episodic or 'binge' drinking in the past 3 months or alcohol-related harms (blackout/injury/ED visit/vomiting) in the past 12 months.
c. For the survey question, "In the last three months, how many days did you use marijuana?" Those screened as negative for the past 12 months of cannabis use were assigned 0.
d. The participants were administered S2BI questions on past-year cannabis use at baseline and past 6-month cannabis use at 6- and 12-month follow-up surveys. The responses were dichotomized as "Any" vs. "Never" cannabis use.
e. The participants were administered S2BI questions on past-year cigarette and e-cigarette use at baseline and past 6-month cigarette and e-cigarette use at 6- and 12-month follow-up surveys. The responses were aggregated and dichotomized as "Any" vs. "Never" nicotine use.

© 2024 Weitzman ER et al. JAMA Network Open.

eTable 7. Intervention Effects Stratified by High-Risk Alcohol Use Behavior at Baseline, Adjusting for Cannabis and Nicotine Use

Outcome	Intervention Effects [a, b]			P values for High-vs. No use/Low-risk Group Interaction
	Exponentiated (ARRR)	(95% CI)	P values	
**Alcohol Use Frequency in Past 3 months**				
**High-risk Alcohol Use at Baseline (n=52)[c] (Time x Intervention)**	0.60	(0.38 to 0.94)	0.03	**0.02**
**No or Low-risk Alcohol Use at Baseline (n=399) (Time x Intervention)**	1.20	(0.85 to 1.69)	0.29	

Abbreviation: ARRR, adjusted relative rate ratios; ED, Emergency Department

a. The intervention effect compares the difference in the changes in the past 3-month alcohol use frequency in days over time. The outcome, past 3-month alcohol use frequency in days, was measured at baseline, 6 months (timepoint 1), and 12 months (timepoint 2) after the randomization.
b. All the models include 3-way interaction between time, intervention and high vs. no or low-risk use groups and adjusted for the baseline demographics, including parental education (less than college vs. college or higher), participants' grades (middle/high school vs. after high school [college or vocational school]), baseline cannabis and nicotine use status (any vs. never).
c. High-risk alcohol use behavior was defined as self-reporting any of the following at baseline: heavy episodic or 'binge' drinking in the past 3 months or alcohol-related harms (blackout/injury/ED visit/vomiting) in the past 12 months.

© 2024 Weitzman ER et al. JAMA Network Open.

eTable 8. Cannabis and Nicotine Use by Intervention Effects and High-Risk Alcohol Use Behavior at Baseline, (N=451)

Outcome	Intervention Effects[a]			P values for High-vs. Low-risk/No-risk Group Interaction
**Past 3-month Cannabis Use Frequency**	**Exponentiated (ARRR)**	**(95% CI)**	**P values**	
**High-risk Alcohol Use at Baseline (n=52)[b] (Time x Intervention)**	0.57	(0.26 to 1.27)	0.17	0.52
**No or Low-risk Alcohol Use at Baseline (n=399) (Time x Intervention)**	0.78	(0.47 to 1.31)	0.35	
**Past 6-month Nicotine Use**	**Intervention Effects[c]**			
	**Exponentiated (AOR)**	**(95% CI)**	**P values**	
**High-risk Alcohol Use at Baseline (n=52)[b] (Time x Intervention)**	0.40	(0.09 to 1.65)	0.20	0.26
**No or Low-risk Alcohol Use at Baseline (n=399) (Time x Intervention)**	0.98	(0.51 to 1.86)	0.94	

Abbreviation. ARRR, adjusted relative rate ratio; AOR, Adjusted Odds Ratio; TAU, Treatment as Usual

a. The intervention effect compares the difference in the changes in the past 3-month cannabis use frequency in days over time
b. High-risk alcohol use behavior was defined as self-reporting any of the following at baseline: heavy episodic or ‘binge’ drinking in the past 3 months or alcohol-related harms (blackout/injury/ED visit/vomiting) in the past 12 months.
c. The intervention effect compares the difference in the changes in the odds of past 6-month nicotine use over time

© 2024 Weitzman ER et al. JAMA Network Open.

eMethods

Intervention

The patient-centered intervention, TGC, was developed incorporating results from qualitative semi-structured interviews among youth with chronic conditions in a pilot study. Adolescents aged 16 to 19 years with a mix of genders, disease types, and histories of substance use were recruited from an existing cohort study.[1] Grounded in social cognitive theories of risk-taking among youth with chronic conditions, the interview covered socio-emotional, situational, and clinical factors affecting alcohol use, as well as patients' acceptability of clinical communication on substance use.

The psychoeducational interventions focused on the unique health impacts of alcohol use on the treatment processes for T1DM, JIA/SLE, and IBD. The tailored content of the intervention was designed to emotionally connect with the health risks of alcohol use for youth with each specific medical condition, while considering the social developmental context of adolescence and providing information about the biological effects of alcohol.

Disease-Specific Information in the Psychoeducational Interventions

All of the slides begin by asking how each condition may have impacted the lives of youths. The slides adopt youth-friendly language throughout, incorporate the perspectives of youths, and address their condition-specific socio-emotional and situational concerns. The slides for each type of condition also explained how drinking can increase the risk of developing or exacerbating depression.

Psychoeducational Contents for Youth with T1DM

The slides of the alcohol health risk section begin by explaining the effects of alcohol on glucose metabolism, how drinking can make patients vulnerable to hypoglycemia or diabetic ketoacidosis, and how these conditions can be mistaken for intoxication, leading to serious consequences.

Psychoeducational Contents for Youth with Rheumatoid Diseases

© 2024 Weitzman ER et al. JAMA Network Open.

The slides in the alcohol health risk section begin by explaining the effects of alcohol on the body, particularly the risk of dehydration, and the health risks associated with the interaction of alcohol and medication.

Psychoeducational Contents for Youth with IBD

The slides in the alcohol health risk section begin by explaining the effects of alcohol on the gut microbiome, the risk of causing inflammation, and worsening gastrointestinal symptoms. They also explain that the use of alcohol can interfere with the metabolism of IBD medications, leading to increased severe adverse events or loss of efficacy.

Measures

Alcohol Risk Intolerance

Alcohol risk intolerance was measured using a 6-point Likert scale to assess individuals' perceived riskiness of consuming different quantities of alcohol. Participants were asked, "How many drinks on one occasion would you consider risky or dangerous drinking for yourself?" The response options included: any, >1, >2, >3, >4, >5, or do not consider alcohol risky or dangerous. The responses were then reverse-coded on a scale from 0 to 5. A response of ">5 or do not consider alcohol risky or dangerous" was coded as 0, while "any number of drinks is risky or dangerous" was coded as 5. Therefore, a higher score on this scale indicates a greater awareness of the risks associated with alcohol consumption.

Adolescent High-Risk Alcohol Use at Baseline

Adolescent high-risk alcohol use at baseline was defined as self-report of heavy episodic or 'binge' drinking in the past 3-month or any alcohol-related harm adapted from the Personal Experience Screening Questionnaire (PESQ)[2] in the past 12 months: "In the past 12 months, how often did you have blackouts while drinking alcohol, when you couldn't remember afterward what had happened?", "How often have you been injured during or after drinking alcohol?", "How often have you vomited

© 2024 Weitzman ER et al. JAMA Network Open.

or thrown up from alcohol use?", "How often have you gone to the emergency room because of problems related to your alcohol use?" (Never, Once or twice, Sometimes, Often). Responses to these questions were dichotomized to any vs. no harm for analysis. Participants who reported any baseline harm or episodic 'binge' alcohol use were defined as high-risk. Three participants who reported either harm or episodic 'binge' alcohol use while missing the other, were included in the no or low-risk use group.

Heavy Episodic or 'Binge' Alcohol Use

Self-report of heavy episodic or 'binge' alcohol use was defined using established age and sex cutoffs.[3] Participant self-identified gender was collected and used as a substitute for the sex criteria for defining excessive drinking, and if the response for gender was missing (n=1) or the response was "other" (n=4), the biological sex recorded in the patient's chart was used. Overall, the agreement between the gender information used in this analysis and the biological sex information recorded in the study enrollment log was kappa 0.96 (95% CI: 0.94-0.98), with 1.8% (8/451) reporting a gender different from their sex categories.

© 2024 Weitzman ER et al. JAMA Network Open.

eReferences

1. Weitzman ER, Wisk LE, Minegishi M, et al. Effects of a Patient-Centered Intervention to Reduce Alcohol Use Among Youth With Chronic Medical Conditions. *J Adolesc Health*. 2022;71(4S):S24-S33. doi:10.1016/J.JADOHEALTH.2021.10.017
2. Winters KC. Development of an adolescent alcohol and other drug abuse screening scale: Personal experience screening questionnaire. *Addictive Behaviors*. 1992;17(5):479-490. doi:10.1016/0306-4603(92)90008-j
3. Donovan JE. Estimated blood alcohol concentrations for child and adolescent drinking and their implications for screening instruments. *Pediatrics*. 2009;123(6). doi:10.1542/peds.2008-0027

© 2024 Weitzman ER et al. JAMA Network Open.
