# Supplementary material for: Disease-Tailored Brief Intervention for Alcohol Use Among Youths With Chronic Medical Conditions: A Secondary Analysis of a Randomized Clinical Trial
Source: JAMA Netw Open. 2024 Jul 10;7(7):e2419858. doi: 10.1001/jamanetworkopen.2024.19858 (PMC11238030; doi:10.1001/jamanetworkopen.2024.19858)
Supplement: Supplement 3. — Data Sharing Statement [file jamanetwopen-e2419858-s003.pdf]

Data Sharing Statement

Weitzman. Disease-Tailored Brief Intervention for Alcohol Use Among Youths With Chronic Medical Conditions. *JAMA Netw Open*. Published July 10, 2024.
doi:10.1001/jamanetworkopen.2024.19858

Data
Data available: No

Additional Information
Explanation for why data not available: Investigators may contact the corresponding author regarding requests to access the data.
